# Genetic vulnerability to *DUSP22* promoter hypermethylation is involved in the relation between in utero famine exposure and schizophrenia

**DOI:** 10.1038/s41537-018-0058-4

**Published:** 2018-08-21

**Authors:** M. P. Boks, L. C. Houtepen, Z. Xu, Y. He, G. Ursini, A. X. Maihofer, P. Rajarajan, Q. Yu, H. Xu, Y. Wu, S. Wang, J. P. Shi, H. E. Hulshoff Pol, E. Strengman, B. P. F. Rutten, A. E. Jaffe, J. E. Kleinman, D. G. Baker, E. M. Hol, S. Akbarian, C. M. Nievergelt, L. D. De Witte, C. H. Vinkers, D. R. Weinberger, J. Yu, R. S. Kahn

**Affiliations:** 1 Brain Center Rudolf Magnus, Department of Psychiatry, University Medical Center Utrecht, Utrecht, The Netherlands; 2 Lieber Institute for Brain Development, Johns Hopkins Medical Campus, Baltimore, USA; 3 Department of Psychiatry, University of California, La Jolla, San Diego, CA USA; 4 VA Center of Excellence for Stress and Mental Health, San Diego, CA USA; 5 Veterans Affairs San Diego Healthcare System, San Diego, CA USA; 6 Departments of Psychiatry and Neuroscience, Icahn School of Medicine at Mount Sinai, New York, USA; 7 Department of Epidemiology and Health Statistics, School of Public Health, Jilin University, Changchun, China; 8 Molecular Pathology, Department of Pathology, University Medical Center Utrecht, Utrecht, The Netherlands; 9 School for Mental Health and Neuroscience, Department of Psychiatry and Neuropsychology, Maastricht University Medical Centre, Maastricht, The Netherlands; 10 Brain Center Rudolf Magnus, Department of Translational Neuroscience, University Medical Center Utrecht, Utrecht, The Netherlands

## Abstract

Epigenetic changes may account for the doubled risk to develop schizophrenia in individuals exposed to famine in utero. We therefore investigated DNA methylation in a unique sample of patients and healthy individuals conceived during the great famine in China. Subsequently, we examined two case-control samples without famine exposure in whole blood and brain tissue. To shed light on the causality of the relation between famine exposure and DNA methylation, we exposed human fibroblasts to nutritional deprivation. In the famine-exposed schizophrenia patients, we found significant hypermethylation of the dual specificity phosphatase 22 (*DUSP22*) gene promoter (Chr6:291687-293285) (*N* = 153, *p* = 0.01). In this sample, *DUSP22* methylation was also significantly higher in patients independent of famine exposure (*p* = 0.025), suggesting that hypermethylation of *DUSP22* is also more generally involved in schizophrenia risk. Similarly, *DUSP22* methylation was also higher in two separate case-control samples not exposed to famine using DNA from whole blood (*N* = 64, *p* = 0.03) and postmortem brains (*N* = 214, *p* = 0.007). *DUSP22* methylation showed strong genetic regulation across chromosomes by a region on chromosome 16 which was consistent with new 3D genome interaction data. The presence of a direct link between famine and *DUSP22* transcription was supported by data from cultured human fibroblasts that showed increased methylation (*p* = 0.048) and expression (*p* = 0.019) in response to nutritional deprivation (*N* = 10). These results highlight an epigenetic locus that is genetically regulated across chromosomes and that is involved in the response to early-life exposure to famine and that is relevant for a major psychiatric disorder.

## Introduction

Schizophrenia is a severe psychiatric disorder with a global life-time risk of around 1% and a typical onset in late adolescence and early adulthood. In addition to a pronounced polygenic component,^1^ several environmental risk factors have been identified,^2^ of which prenatal famine is one of the strongest: an almost two-fold increase was reported in offspring conceived during the Dutch hunger winter in 1945^3^ and at the time of the Great Chinese famine (1959–1961).^4–6^

The mechanism underlying the relationship between famine exposure and schizophrenia risk remains unclear, but emerging evidence suggests that epigenetic reprogramming in response to famine exposure may play a role. Indeed, famine exposure in the first trimester of pregnancy leads to DNA methylation changes and these in turn have been found to be related to cardiovascular disorders.^7–11^ However, the relationship between famine-induced epigenetic changes and schizophrenia has not been studied.

We hypothesized that changes in DNA methylation play a role in the increased risk to develop schizophrenia after in utero exposure to famine. To test this hypothesis, we focused on the great famine in China between 1959 and 1961, which led to an estimated death toll of over 30 million.^12^ The high penetrance of famine in a large rural population during a restricted period offers an opportunity for selective sampling of schizophrenia patients and healthy controls on the basis of their exposure to famine. We also studied the role of the identified DNA methylation marks in blood and brain DNA samples of unexposed schizophrenia patients and controls. Moreover, we carried out in vitro experimental studies whereby human fibroblasts were exposed to nutritional deprivation to directly investigate methylation responses to nutritional deprivation without the potential confounds of genetic differences, medication, and other environmental factors.

Contemporary studies of DNA methylation show that much of the variability in DNA methylation is controlled by genetic variation in the same genetic region (in *cis*)^13^ as well as in genetic regions more distant from the methylation mark (in *trans*).^14^ Another point of increasing interest is the relation of the DNA methylation differences with gene expression, as a relevant functional readout of methylation differences.^15^ Further analyses therefore investigated the relationship of identified methylation differences with genotypes as well as their relations with expression.

## Results

Four samples were included in the current paper. (1) The Chinese famine sample in which the relation between famine and schizophrenia was studied. (2) A case-control blood sample from the Netherlands in which we analyzed differences between unexposed schizophrenia patients and unaffected individuals using DNA from whole blood. (3) The case-control brain sample in which we replicated case-control differences using DNA from brain tissue and analyzed the relations with genotype and gene expression. (4) Fibroblasts cultures obtained from schizophrenia patients and healthy controls. Table 1 gives an overview of the characteristics of these four samples.

**Table 1:** Sample characteristics of study samples

	Sample 1: Chinese famine		Sample 2: Case-control blood		Sample 3: Case-control brain		Sample 4: Fibroblasts	
	Schizophrenia	Controls	Schizophrenia	Controls	Schizophrenia	Controls	Schizophrenia	Controls
*N*	74	79	15	49	91	123	5	5
Male (%)	46 (62%)	31 (39%)	9 (60%)	4 (14%)	55%	67%	3 (60%)	2 (40%)
Mean age (sd)	47.3 (0.7)	47.9 (0.8)	40.1 (13.8)	35.9 (17.0)	52.6 (5.2)	45.9 (16.8)	39.0 (10.3)	36.5 (6.5)
Famine exposure (%)	23 (31%)	25 (32%)	–	–	–	–	In vitro	In vitro
Tissue source	Blood		Blood		Brain (DLPFC)		Fibroblast culture	
Methylation	450K BeadChip array		450K BeadChip array		450K BeadChip array		EPIC BeadChip array	
Reference workflow	–		–		Jaffe et al.^4^		–	

*SCZ* schizophrenia, *DLPFC* dorsolateral prefrontal cortex

**Fig. 1:**
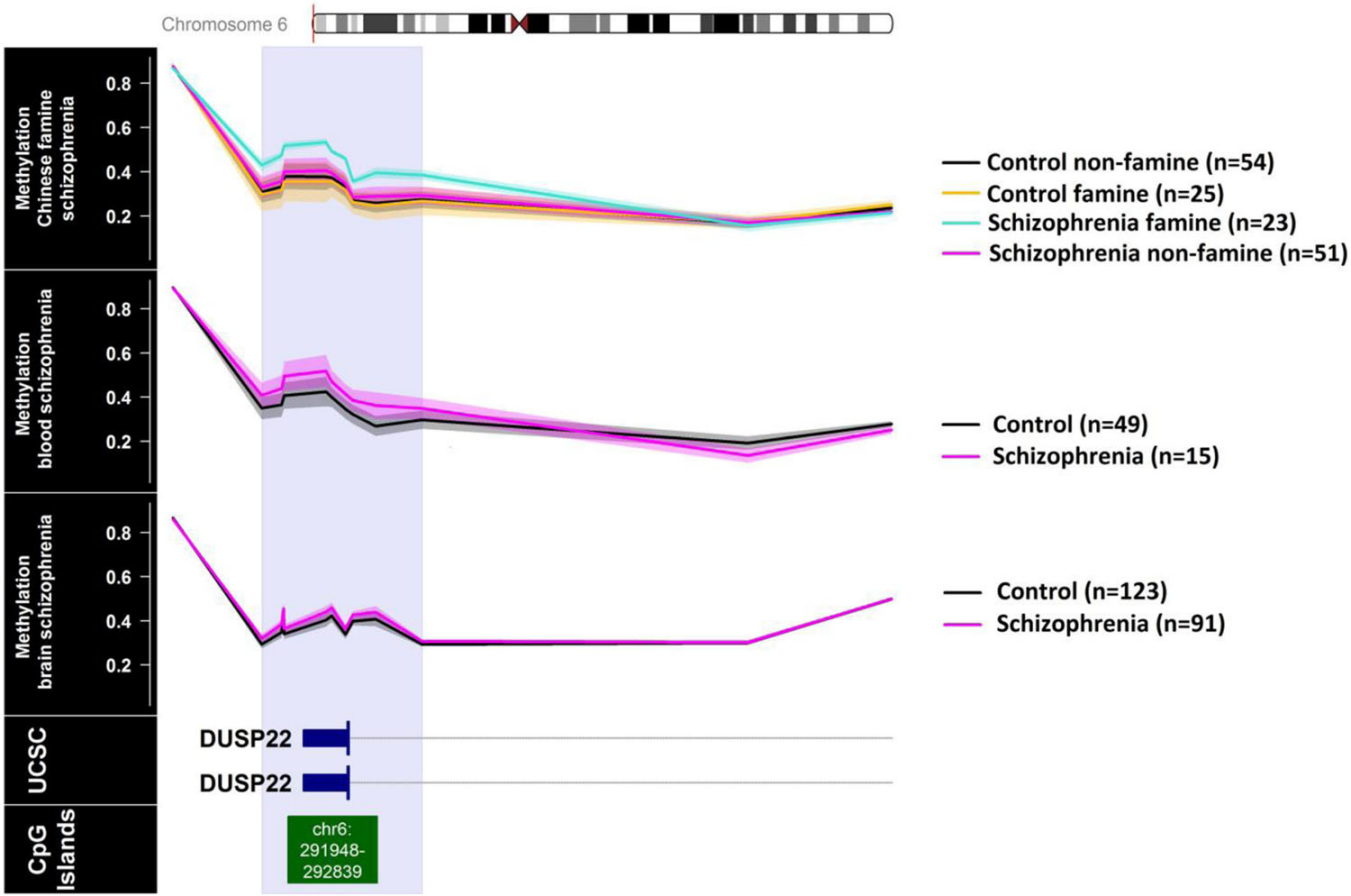
Identification and replication of a differentially methylated region in *DUSP22* in blood. Overview of the 3000 bp area downstream and upstream of the dual specificity phosphatase 22 (*DUSP22*) differentially methylated region (DMR). The top panel displays the blood DNA methylation levels per group in the Chinese famine discovery sample (first panel). The second and third panels contain the DNA methylation levels for schizophrenia patients and healthy controls in blood or brain tissue, respectively. The other panels indicate the presence of coding exons (blue blocks) and non-coding introns (gray line) of the *DUSP22* gene (fourth panel), and the location of a CpG island (fifth panel) based on information extracted for genome build Hg19 from the UCSC website41 with the gviz R package 42. The *DUSP22* DMR is indicated across all panels with a light-blue rectangle; chr, chromosome

The genome-wide methylation analysis of the Chinese famine sample identified one single region containing the dual specificity phosphatase 22 (*DUSP22)* gene promoter with higher DNA methylation levels in the famine-exposed schizophrenia patients compared to all other groups (Chr6: 291687-293285, Family Wise Error Rate (FWER) = 0.01). *DUSP22* methylation was also significantly higher in Chinese schizophrenia patients independent of famine exposure (*B* = 0.07, *p* = 0.025). *DUSP22* hypermethylation in the same region was also significant in blood DNA samples of Dutch schizophrenia patients (*N* = 15, mean methylation = 0.43, sd = 0.10) compared to healthy controls (*N* = 49, mean methylation = 0.32, sd = 0.20, *p* = 0.03). *DUSP22* methylation was also higher in postmortem prefrontal cortex (PFC) tissue of schizophrenia patients (*N* = 91; mean methylation = 0.40, sd = 0.10) compared to unaffected controls (*N* = 123; mean methylation = 0.37, sd = 0.13) (*B* = 0.35, *p* = 0.007). Figure 1 and Table 2 show the results from the association analysis.

**Table 2:** Detailed data by exposure and schizophrenia status

Chinese famine cohort				
	Schizophrenia		Controls	
	Exposed	Unexposed	Exposed	Unexposed
*N*	23	51	25	54
Age	50.1 (0.6)	46.7 (0.8)	50.3 (0.5)	46.8 (1.0)
Male (%)	18 (69%)	28 (54%)	10 (40%)	21 (39%)
*DUSP22* Methylation (sd)	0.46 (0.04)	0.35 (0.17)	0.31 (0.19)	0.33 (0.18)

Case-control blood cohort		
	Schizophrenia	Controls
*N*	15	49
Age, mean (sd)	40.1 (13.8)	35.9 (17.0)
Male (%)	60%	10%
*DUSP22* Methylation (sd)	0.43 (0.10)	0.32 (0.20)

Case-control brain cohort		
	Schizophrenia	Controls
*N*	91	123
Age, mean (sd)	52.6 (5.2)	45.9 (16.8)
Male (%)	55%	67%
*DUSP22* Methylation (sd)	0.40 (0.10)	0.37 (0.13)

### Genetic control of *DUSP22* DNA methylation

**Fig. 2:**
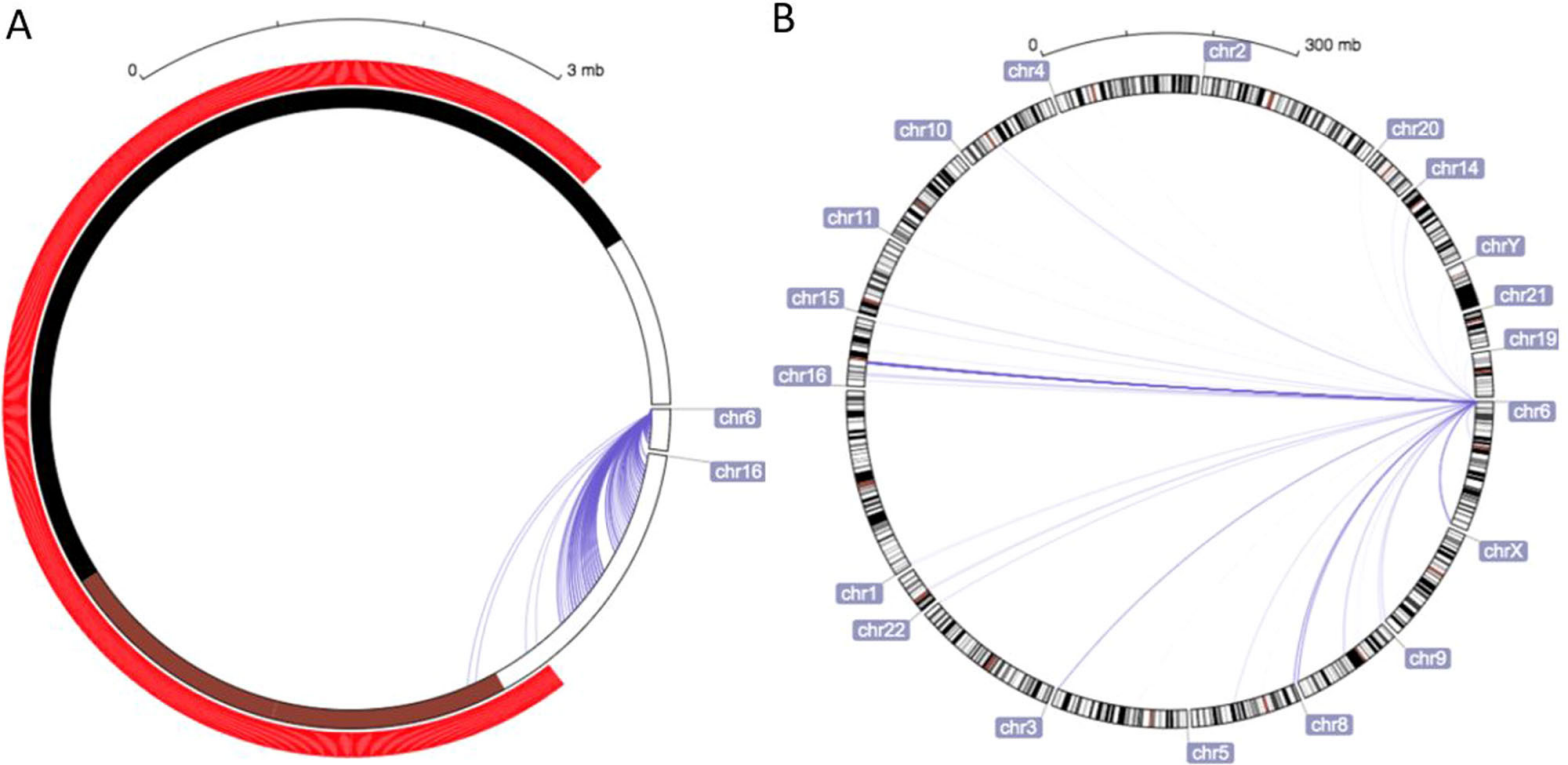
Overview of the chromosome–chromosome interactions measured with in situ Hi-C. Panel **a** zooms into the *DUSP22* DMR, while **b** provides an overview of the chromosome interactions. A darker blue indicates more frequent interactions

**Fig. 3:**
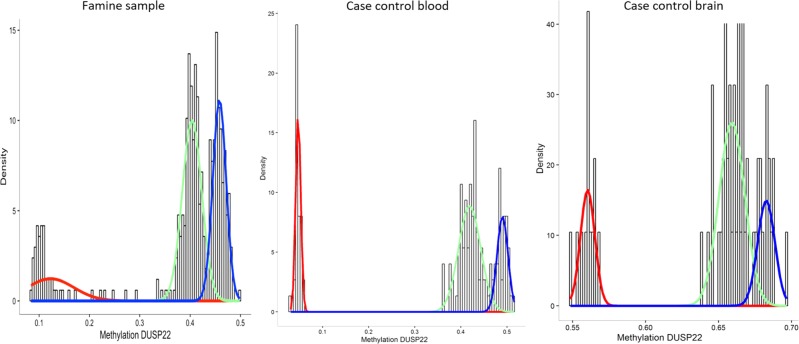
Density plot of the average methylation at the *DUSP22* differentially methylated region (DMR) in the four population samples: the Chinese famine sample, the case-control blood sample, the blood genomics sample, and the brain case-control sample. The colored lines represent the estimation of the underlying distributions in the respective samples

In the brain case-control sample, the association between genetic loci and methylation levels at the ten loci (CpGs) in the *DUSP22* differentially methylated region (DMR) was examined for 7.5 million observed + imputed single nucleotide polymorphisms (SNPs) with minor allele frequency (MAF >5%). We identified 69 SNPs, all on chromosome 16, that were associated with all ten methylation loci at the *p* < 10^–20^ significance level (see supplementary table 1). This genetic regulation outside the *DUSP22* region (in *trans*) is consistent with previous studies^14^ and three online databases of genetic variants associated with methylation methylation Quantitative Trait Locus (mQTLs) for either fetal brain tissue (http://epigenetics.essex.ac.uk/mQTL/^16^) or whole blood (http://genenetwork.nl/biosqtlbrowser/^17^and http://www.mqtldb.org/^18^), reporting five *trans*-SNPs on chromosome 16 (rs1433753, rs9674439, rs12923277, rs12927233, and rs12933929). The underlying genetic background spans a region on chromosome 16 (Chr16:34190042-46441560) as large as 12 Mb, and the previously reported five *trans*-SNPs are at least 15,000 base pairs away from each other. It is worth noticing that data on chromosome interactions (obtained using Hybridization Capture—Hi-C) in postmortem neurons confirm the strong interaction between the *DUSP22* DMR and the mQTL region on chromosome 6. The strongest interaction is present between the 3′ end on chromosome 16 (chr16:34160000-34200000) and the region chr6:280000-320000 (see Fig. 2a, b). Visual inspection of the data of Rao et al.^19^ from human GM12878 B-lymphoblastoid cells also indicates that the entire region containing these *trans*-SNPs is in interchromosomal contact with the *DUSP22* promoter region (see supplemental data 1). Subsequent mixture analysis identified three underlying Gaussian distributions in the samples of the study (see Fig. 3) that we used as an indicator of genetic determinant of the methylation levels, subsequently coined “genetic background”.

### Influence of genotype on schizophrenia risk

The Chinese famine sample showed a significant gene–environment interaction between the genetic background and famine exposure on schizophrenia risk (*B* = 1.20, *p* = 0.042). After adjustment for genetic background, the relationship of *DUSP22* methylation with famine and schizophrenia was only present in a selection of participants without the 35 participants with a genetic predisposition for low invariable methylation levels (*N* = 118, *B* = 0.028, *p* = 0.072). In the Dutch case-control sample, the association between *DUSP22* promoter methylation and schizophrenia persisted after adjustment for genetic background (*B* = 0.741, *p* = 0.025). In the postmortem brains, the association between *DUSP22* promoter methylation and schizophrenia was attenuated after adjustment for genetic background (*B* = 0.13, *t* = 1.745, *p* = 0.083).

### Ethnicity

Ethnicity influenced the relationship between *DUSP22* methylation and schizophrenia in the brain case-control sample (Schizophrenia by Race interaction: *B* = 0.680, *p* = 0.007). The genetic background of *DUSP22* methylation was significantly different between African–American and Caucasian subjects (*p* < 0.001 in Pearson’s *χ*^2^ test). The absence of the low methylation genotype in the African–Americans contributes to the lower variation in *DUSP22* DMR methylation levels (see Table 3). This is in line with the stratified analyses for ethnicity that show an association between *DUSP22* methylation and schizophrenia in the Caucasians only (*B* = 0.692, *p* = 0.002), and not in the African–Americans (*B* = −0.086, *p* = 0.436).

**Table 3:** Sample characteristics of the brain dataset per ethnicity

	African–American	Caucasian	All
*N*	99	115	214
Age (mean (sd))	49.24 (16.30)	48.28 (16.62)	48.73 (16.44)
Male (%)	58 (58.6)	74 (64.3)	132 (61.7)
Schizophrenia (%)	38 (38.4)	53 (46.1)	91 (42.5)
*DUSP22* DMR (mean (sd))	0.43 (0.07)	0.34 (0.14)*	0.38 (0.12)
*DUSP22* expression (mean (sd))	2.83 (0.33)	2.81 (0.33)	2.82 (0.33)
Genetic background (%)			
1	3 (3.1)	27 (23.5)*	30 (14.0)
2	35 (36.4)	65 (56.5)*	100 (46.7)
3	61 (63.5)	23 (20.0)*	84 (39.3)

*p < 0.001 in either a Pearson’s *χ*^2^ test (for genotype) or *t*-test (for methylation levels)

### Smoking and urban background

In the Chinese famine sample, as expected, smoking was more frequent in the schizophrenia patients (*B* = 0.01, *p* = 0.046). No differences between the famine and non-famine groups were present (*p* = 0.9). The *DUSP22* promoter methylation was also not associated with the smoking proxy (*B* = 0.028, *p* = 0.112), nor is it highlighted in previous association studies into smoking.^20,21^ Also inclusion of urban background in the model did not alter the results. In the case-control blood samples, five of 15 schizophrenia patients were current smokers in contrast to three of 49 smokers in the healthy controls (*χ*^2^ test, *p* = 0.02), *DUSP22* promoter methylation was not associated with schizophrenia status (*B* = −0.79, *p* = 0.223). Smoking was dealt with in the case-control brain samples by adjusting the methylation data using the first principle components.^22^ Supplemental data Figs. 1,2, and 4 show the relations of smoking with the variables of interest.

### *DUSP22* methylation and expression

A correlation between *DUSP22* methylation and expression was not present in the brain samples, nor was genetic background associated with *DUSP22* expression. In contrast, the schizophrenia patients had significantly lower *DUSP22* transcript levels (schizophrenia: *N* = 60, *DUSP22* expression = 2.97, sd = 0.17; controls: *N* = 96, *DUSP22* expression = 2.87, sd = 0.21) (*B* = −0.09, *p* = 0.005), also after adjustment for genetic background (*B* = −0.14, *p* < 0.001), suggesting transcriptional regulation of *DUSP22* by other factors than DNA methylation in the adult brain of schizophrenia patients.

### Fibroblast

Depriving fibroblasts from nutrition by withholding 15% fetal bovine serum (FBS) resulted in a significant increase in *DUSP22* methylation (Paired Wilcoxon, *p*-value = 0.049) and an almost two-fold increase in *DUSP22* expression after 72 h (Paired Wilcoxon, *p*-value = 0.019). Analysis of the methylation difference between the famine and control conditions showed no significant difference in response comparing fibroblasts from schizophrenia patients and controls. Removal of one member of the included healthy homozygous twin pair slightly reduced the significance (*p* = 0.054 or *p* = 0.063 depending on which member was removed).

## Discussion

Genome-wide analysis of DNA methylation in whole blood from a sample of schizophrenia patients (*N* = 74) and controls (*N* = 79), where one-third of both groups were exposed in utero to the great famine in China (1959–1961), identified one region in the promoter of the dual specificity phosphatase 22 (*DUSP22)* gene (Chr6:291687-293285) with significantly higher DNA methylation levels in famine-exposed schizophrenia patients compared to unexposed patients and healthy controls. In this sample, patients also had significant hypermethylation independent of famine exposure suggesting that *DUSP22* hypermethylation is primarily involved in schizophrenia. In an independent but unexposed Dutch sample, a similarly unbiased genome-wide analysis of whole-blood DNA identified the same hypermethylated *DUSP22* region in schizophrenia patients (*N* = 15) as compared to healthy individuals (*N* = 49). In the postmortem tissue from the PFC, significant *DUSP22* hypermethylation was also found in schizophrenia patients (*N* = 91) compared to unaffected individuals (*N* = 123). Support for a direct relationship between famine exposure and *DUSP22* methylation was obtained by depriving the fibroblasts of schizophrenia patients (*N* = 5) and controls (*N* = 5) from nutrition. *DUSP22* methylation and expression significantly increased in response to nutritional deprivation.

The hypermethylated region in *DUSP22* encompasses a CpG island in the promoter as well as the histone marks H3K27ac and H3K4me3 that are indicative of active gene transcription in both PFC brain and blood cells.^23,24^ This region contains ten CpG loci and showed clustering (banding) of the methylation levels indicative of genetic regulation. In the postmortem brains, we identified 69 SNPs on chromosome 16 with highly significant associations with *DUSP22* methylation, which included previously reported mQTLs in blood^17^ and brain.^16^ This interaction between the *DUSP22* DMR on chromosome 6 and the SNPs on chromosome 16 (Chr16:34190042-46441560) is consistent with new data of Hi-C proximity maps from human postmortem brains as well as previously published Hi-C data on human lymphoblastoid cells.^19^ These data highlight the importance of SNPs that physically interact in three-dimensions (3D) with chromatin and influence target transcript levels.^14^ The identified mQTLs are not associated with schizophrenia in the most recent GWAS meta-analysis^4^ suggesting that these genetic variants are not primary risk alleles for schizophrenia.

The biological relevance of *DUSP22* methylation could not be substantiated by a correlation between *DUSP22* methylation and expression in the brain samples, nor was genetic background associated with *DUSP22* expression. In the brain samples, the schizophrenia patients had significantly lower *DUSP22* transcript levels uncorrelated with *DUSP22* methylation and genetic background suggesting that in the adult brain of schizophrenia patients, transcriptional regulation of *DUSP22* is independent of DNA methylation or that other factors interfere with the relationship between *DUSP22* methylation and expression. There are several factors that may confound the reported relationships, and although we provided replication and have investigated several potential confounders including smoking, the relatively small sample sizes and residual confounding remain a limitation. Also urban origin was defined by the current dwelling, and it is possible that it is not an accurate reflection of the birthplace.

The results of this series of experiments suggest that altered transcriptional regulation of *DUSP22* in response to famine is a schizophrenia susceptibility factor. *DUSP22* is a recently identified Dual Specificity Phosphatase. Studies on the hippocampus of patients with Alzheimer’s disease have linked promoter hypermethylation of *DUSP22* to changes in TAU phosphorylation,^25^ which in turn has been connected to nutritional deprivation.^26^ A further link between *DUSP22* methylation and nutrition is supported by trial data showing that low *DUSP22* DNA methylation at baseline predicted high weight loss in response to a dietary intervention.^27^

Notwithstanding these pre-existing links between *DUSP22* methylation and nutrition, the presence of higher *DUSP22* methylation in the blood and brain of schizophrenia patients not exposed to famine also suggest that aberrant *DUSP22* methylation is more generally involved in the neurodevelopmental processes underlying the etiology of schizophrenia. The absence of any signal of *DUSP22* methylation in recent large EWAS studies in schizophrenia^28–32^ and the absence of an association with the genetic variation associated with these methylation differences^4^ points out that *DUSP22* is not a primary risk gene for schizophrenia. Instead the evidence from this and other studies point to a role of *DUSP22* methylation in regulating responses to a variety of environmental stressors^33–36^ that may deviate early developmental processes of the brain.^37,52^ It is possible that such vulnerability in combination with particular environmental insults increase the risk of schizophrenia in a subgroup. In support, a recent study by Vitale et al.^38^ showed that one of the CpG in our DMR (cg11235426, Chr6: 292522) was differentially methylated in induced pluripotent stem cells (iPSCs) from schizophrenia patients as compared to controls (logFC = −2.44, FDR = 0.04) and in schizophrenia patients prenatally exposed to diethylstilbestrol (DES), *DUSP22* methylation (Chr6:291687-293331) was higher compared to exposed controls (unadjusted *p*-value = 0.00018).^39^

Further, of note is the strong *trans* genetic regulation that stretches over 30 Mb on a different chromosome that coincides with chromosome-chromosome interactions.^19^ The data fit a model where genetic background determines environmental susceptibility of the *DUSP22* gene and schizophrenia risk. Famine changes the epigenetic regulation of the *DUSP22* promoter in those that are genetically vulnerable. This putative epigenetic mechanism of early-life environmental influences on brain development is likely to be important, not only for understanding the etiology of schizophrenia, but also because it opens new perspectives on mechanisms of gene–environment interactions.

## Material and methods

### Chinese famine sample

The Chinese famine started suddenly in 1959 as the result of agriculture reforms by Mao and lasted until 1961. Whereas the onset was sudden, the exact end dates vary by geographical location due to a subsequent drought that affected the northern provinces.^40^ In collaboration with the University in Changchun in the Northern Province of Jilin, we included schizophrenia patients and healthy controls that had been exposed to famine within the first 3 months of gestation based on a birth date between January 1960 and September 1961. All participants gave written informed consent. In order to balance the potential influence of urban background,^5^ recruitment was stratified for rural and city hospitals. A total of 74 schizophrenia patients and 81 healthy controls approximately matched for famine exposure were assessed. Information on medication was obtained using a structured interview. Diagnosis was made using a full psychiatric evaluation according to DSM IV criteria by licensed psychiatrists. Patients diagnosed with schizophrenia according to DSM IV included those with 295.*x* Schizophrenia, 295.4 Schizophreniform disorder, 295.7 Schizoaffective disorder, 297.1 Delusional disorder, but excluded 298.8 Brief psychotic disorder, 297.3 Shared psychotic disorder, 293.*x* Psychotic disorder due to a medical condition, 293.*x* Substance-induced psychotic disorders, and 298.9 Psychotic disorder not otherwise specified. The absence of a mental health disorder in the healthy controls was assessed by a Chinese translation of the Mental Health Screening Form-III (MHSF-III).^41,42^ If more than 10% of the patients used a specific medication type, we investigated its association with DNA methylation (using the first principal component of the methylation measures). Based on this criterion, clozapine (*n* = 37) and chlorpromazine (*n* = 26) use were examined as potential confounders in the Chinese famine discovery sample by analyzing the correlation with main determinants (famine and diagnosis) and outcome (methylation). Smoking was addressed similarly as to Hannon et al.^43^ whereby a smoking proxy was calculated based on DNA methylation values for CpGs previously associated with smoking.^20,21^ In cases where these correlations were significant and changes in the coefficient were larger than 10%, the variable was considered a potential confounder and models were adjusted by including it as covariate (see Extended Data Fig. 1). Based on their correlation with general DNA methylation levels (Extended Data Fig. 2), covariates included: age, gender, the first two DNA methylation-based ancestry principal components as well as the cell-type proportion estimates based on the Houseman algorithm.^44^ We separately investigated the potential role of urban versus rural background by including this as covariate in the analysis. We excluded one sample based on gender mismatch with the gender prediction from DNA methylation levels (one non-famine-exposed healthy control) and one sample based on the use of insulin (one famine-exposed healthy control).

### Case-control blood samples

Whole-blood DNA samples were collected in 2016 from 15 schizophrenia patients (9 male, mean age = 40.1, sd = 13.8) and 49 healthy controls (4 male, mean age = 35.9, sd = 17.0) at the University Medical Center, Utrecht, The Netherlands. Participants were of Dutch origin with three or more Dutch grandparents. Patients were outpatients from the University Medical Center. Eligibility was assessed by their treating psychiatrist and inclusion was done by research staff (LH). Healthy controls were recruited in the general population using online and paper advertisements. All participants gave written informed consent. Diagnosis of schizophrenia (295.*x*) (*N* = 11) or schizoaffective disorder (295.7) (*N* = 4) according to DSM IV criteria was verified in medical records, supplied by the treating physician or established with the Structured Clinical Interview for DSM-IV (SCID).^45^ In the healthy controls, the absence of a DSM-IV diagnosis was assessed with the Mini International Neuropsychiatric Interview (MINI) plus^46^ by at least one well-trained rater. Medication use was collected using a self-report questionnaire. All schizophrenia patients were on a stable (at least 1 month) dosing schedule of psychotropic medication.

### Case-control brain samples

We investigated *DUSP22* DNA methylation, mRNA, and genotype in 214 adult postmortem dorsolateral PFC samples of patients with schizophrenia according to DSM-IV (*N* = 91, 50 male, mean age = 52.6, sd = 5.2) and unaffected controls (*N* = 123, 82 male, mean age = 45.9, sd = 16.8) from the Lieber Institute.^22^ All participants gave written informed consent. A majority was Caucasian (Patients: *N* = 53, 27 male, age = 50.4 ± 15.6; Controls: *N* = 62, 47 male, age = 46.4 ± 17.4) but a sizable proportion (46%) was of Afro-American descent (Patients: *N* = 38, 23 male, age = 55.5 ± 14.2; Controls: *N* = 61, 35 male, age = 45.3 ± 16.4). Postmortem diagnosis according to DSM-IV was obtained when two board-certified psychiatrists reached consensus after reviewing data from as many sources as possible (i.e., multiple psychiatric records, police reports, neuropathology reports, medical examiner’s information, toxicology screen, postmortem family interview).^47^ Medication was assessed via a chart review and/or toxicology on brain tissue.^22^

### Fibroblast cell lines

Fibroblast cell lines were established using skin biopsies from five schizophrenia patients (2 male, mean age = 39.0, sd = 10.3) and five age-matched healthy controls (1 male, mean age = 38.4, sd = 7.0). All participants gave written informed consent. One healthy control donor (control 6) turned out to be the homozygous co-twin of another (control 8). All participants were Dutch and had three or more Dutch grandparents. Schizophrenia (295.*x*) was diagnosed according to DSM-IV as established using the Comprehensive Assessment of Psychiatric symptoms and History (CASH).^48^ The mental health status of healthy controls was checked using the MINIplus interview.^46^

### General analysis

All data were obtained after written, informed consent from all participants and local medical ethical approval. This research was conducted in accordance with all relevant guidelines and procedures, and the work was approved by the University Medical Center medical ethical review board. Statistical analyses were carried out using R version 3.2.3.^49^ For DNA methylation, *β* values (the ratio between methylated and unmethylated probe intensities as a measure of methylation percentage) were used for graphical display and reporting because they are more intuitive, but analyses were carried out using *M*-values (log2 of *β* values), which have better statistical validity,^50^ but give similar results.

### Genome wide analysis of DNA methylation

All details of quality control, batch effect removal and analysis are reported in the supplemental materials. In the Chinese famine and the case-control blood sample, quality control included filtering for detection of *p*-values, low bead count, cross hybridizing- and non-autosomal probes. Normalization was done using functional normalization as implemented in the minfi R package.^51^ Batch effects were limited as a result of the distribution of the samples of the array; we only removed a small remaining batch effect for position. Methylation levels were adjusted for cell-type composition estimates derived using the Houseman algorithm.^44^ Linear regression was used to identify associations of single CpG with famine and schizophrenia and the bumphunter algorithm^52^ for differential methylated regions (details in supplement).

The quality control and analysis of the brain case-control sample were described previously^22^ and include adjustment for neuronal proportion and adjustment for technical batches.

### Banding and *trans* genetic regulation of *DUSP22* methylation

At the *DUSP22* DMR, there were different DNA methylation bands in all four population samples: the Chinese famine sample, the case-control blood sample, the blood genomics sample, and the brain case-control sample (see Fig. 1). Measurement errors due to genetic variants in *cis* that can interfere with hybridization^13,53^ were ruled out by showing that five out of 12 SNPs that are located on the DNA sequence of the *DUSP22* DMR in the dbSNP142 database and that were available in both the MRS and brain case-control samples (rs860102, rs3734780, rs117766562, rs9503164, and rs148619589) had a minor allele frequency (MAF) <5% and were not associated at the 5% level with *DUSP22* DMR methylation in either the blood genomics samples or the brain case-control sample. Therefore, we did not examine these *cis*-SNPs any further. To examine the influence of genetic background on DNA methylation levels, we investigated the association of the mean methylation of the ten CpG in the *DUSP22* DMR with genotype. Moreover, we used finite mixture modeling as implemented in the mixtools R package to derive the underlying genetic background based on the mixture of three distributions in the methylation levels of the *DUSP22* DMR. In all samples, each participant was allocated to one of the three “genetic” groups based on the level of methylation. To investigate the potential confounding influence of genotype, we used membership to the methylation distribution (1,2,3) as ordinal indicator in *χ*^2^ tests, logistic regression, or linear regression as appropriate (see supplement 1). For the stratified analysis by genotype levels (excluding participants with membership to low methylation genetic background), genetic background was not added to the models.

### Analysis of expression in brain and blood

Details of the analysis of the relationship between *DUSP22* methylation and expression of transcript levels can be found in the supplement. In short, we analyzed the association between DNA methylation levels of the *DUSP22* DMR and *DUSP22* expression and adjusted for the underlying genetic background.

### In situ Hi-C data from human postmortem brain tissue

Flash frozen postmortem brain tissue was obtained from the Human Brain Collection Core (HBCC) of the National Institute of Mental Health (NIMH), US. The anterior cingulate cortex sample used in this study is from a 35-year-old non-psychiatric female. Nuclei isolation through extraction, purification, and fluorescence-activated nuclear sorting (FANS) was performed with minor adjustments per Kundakovic et al.^54^ (see supplement).

### Nutritional deprivation of fibroblasts

Details of the procedures and analysis are available in the supplemental materials. In short, fibroblasts from ten donors were cultured in Minimum Essential Medium (MEM) (Gibco®) with or without 15% FBS to mimic famine. DNA and mRNA were extracted and analyzed using EPIC methylation arrays and qPCR. Non-parametric analysis of average methylation of all *DUSP22* CpGs for the paired observations of all ten donors for the 72-h nutritional deprivation compared to the condition with FBS was done using a paired Wilcoxon Signed Rank Test.

## Electronic supplementary material

Supplemental method

Supplemental table 1

## Data Availability

The DNA methylation datasets generated during and/or analyzed during the current study are available in the GEO repository, https://www.ncbi.nlm.nih.gov/geo/query/acc.cgi?acc=GSE116380 Clinical data are not available due to ethical restrictions.

## Electronic supplementary material

**Supplementary information** accompanies the paper on the *npj Schizophrenia* website (10.1038/s41537-018-0058-4).
