# Supplementary material for: Genetic vulnerability to DUSP22 promoter hypermethylation is involved in the relation between in utero famine exposure and schizophrenia
Source: NPJ Schizophr. 2018 Aug 21;4:16. doi: 10.1038/s41537-018-0058-4 (PMC6104043; doi:10.1038/s41537-018-0058-4)
Supplement: Supplementary file 1 — Supplemental method [file 41537_2018_58_MOESM1_ESM.docx]

Supplement to

**Genetic vulnerability to DUSP22 promotor hypermethylation is involved in the relation between in utero famine exposure and schizophrenia**

**Extended methods**

*Genome wide analysis of DNA methylation in the Chinese famine cohort*

DNA was extracted from whole blood using the Gentra Puregene Kit (Qiagen, Valencia, CA, USA). After bisulphite conversion (ZYMO Research, Orange, CA, USA) genome-wide DNA methylation measures were obtained using Illumina Infinium HumanMethylation450K BeadChip arrays. Intensity read outs, beta and M-value calculation and cell-type proportion estimates were obtained using the minfi package (version 1.10.2) in Bioconductor^1^. Probes were excluded based on a bead count less than three (n=279 probes) or a detection p-value larger than 0.001 in at least 5% of the samples (n=2,125 probes). Non-autosomal or cross hybridizing probes^2^ were discarded as were loci with SNPs of Minor Allele Frequency larger than 1 percent within 1 base pairs of the primer^3^. None of the blood samples had over 1% of failed probes. The experiment was conducted such that gender, famine exposure and patient status were equally distributed over the sixteen arrays. However no account was made of row position that has since been implicated in batch effects. We therefore performed careful adjustment for technical batches without compromising the phenotype related differences. To this end, we applied the functional normalization procedure implemented in the minfi package (version 1.10.2)^4^ that uses the control probes of the 450K array to correct for technical artifacts (see Extended Data Fig.2 for heatmap). A small remaining batch effect for position on the array was driven by row placement of one famine exposed healthy control. A sensitivity analysis indicated exclusion of this one subject did not affect the identification of the *DUSP22* differentially methylated region (N=152; FWER=0.02). Therefore in the main article we report the findings in the complete sample including this healthy control (N=153). The coefficients of the association analysis were obtained from the interaction term between maternal famine and schizophrenia status in a linear regression model. Besides the interaction term the linear regression model included maternal famine and schizophrenia status as main effects and, based on their correlation with general DNA methylation levels (Extended Data Fig. 2), included as covariates age, gender, the first two DNA methylation-based ancestry principal components as well as the cell-type proportion estimates based on the Houseman algorithm^5^ (DNA methylation ~ Schizophrenia x Famine + Schizophrenia + Famine + age+ gender + CD4T + CD8T + Mono + NK + BCell). In contrast, clozapine and chlorpromazine were not associated with DNA methylation levels and therefore not added to the regression model (Extended Data Fig. 1). We used this linear regression model to investigate differentially methylated CpG loci, but examination of the QQplot indicated the analysis was underpowered to detect genome-wide differentially methylated probes (see Extended Data Fig. 3). We identified regional differentially methylated regions (DMRs) using the bump hunting algorithm^6^ utilizing the coefficient at each individual locus to identify regions, within a 500bp moving frame, that exceed the 95th percentile of all estimated coefficients genome-wide. Region-level statistics for the DMR were calculated and the family-wise error rate (FWER) was estimated based on 1000 bootstraps under the null.


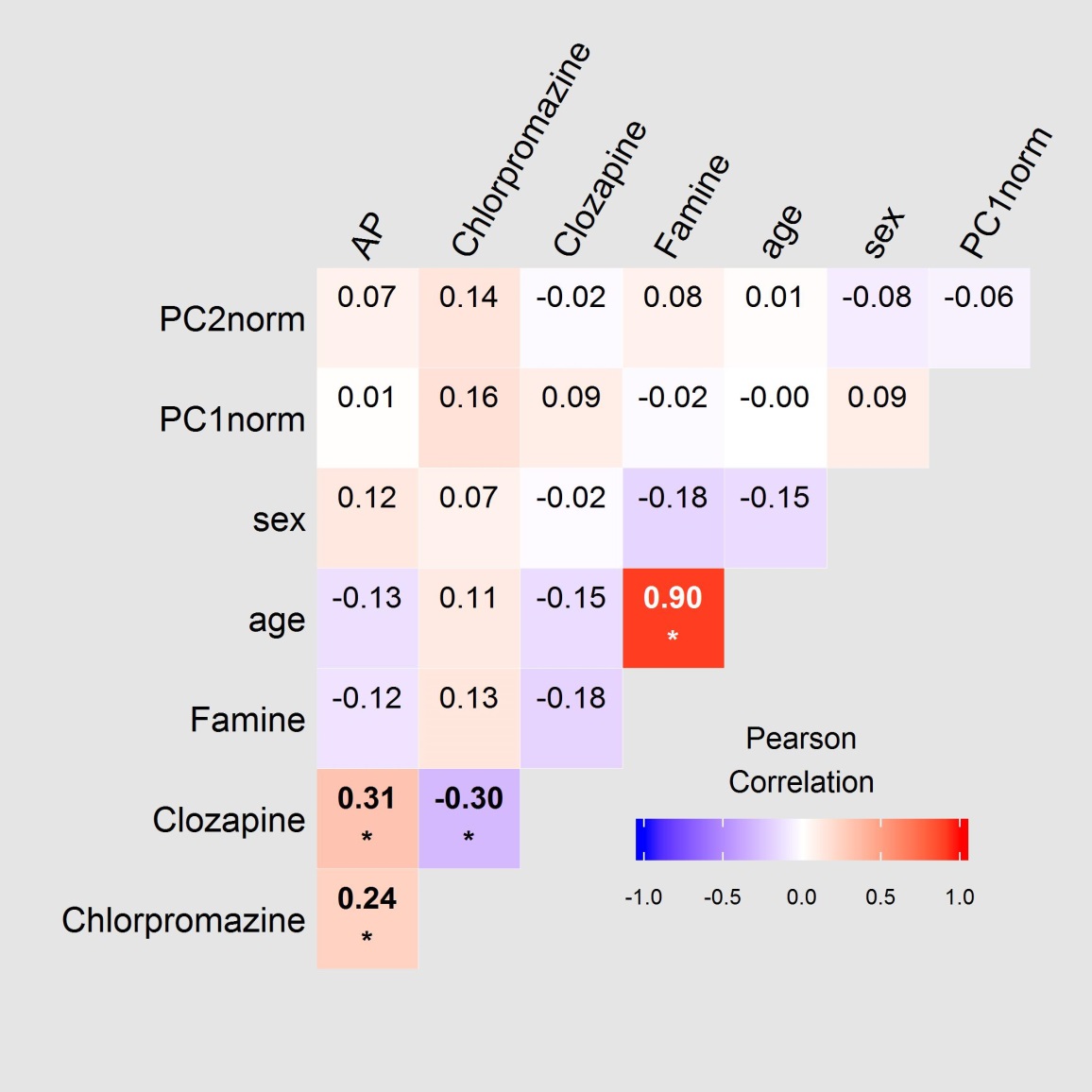


**Extended Data Fig. 1** No correlation between methylation levels (=PC) and medication use (clozapine, chlorpromazine or antipsychotic (=AP)) in the schizophrenia patients of the Chinese famine discovery sample (n=74). Note that medication use is also not associated to famine exposure. Positive correlations are red and negative correlations are blue, the color intensity is related to the size of the correlation coefficient. Significant values are bold and denoted by * p<0.05. Abbreviations: AP=antipsychotic use, PC= principal component. The mention of ‘norm’ after the principal component number (e.g. PC1norm), stands for after functional normalization.


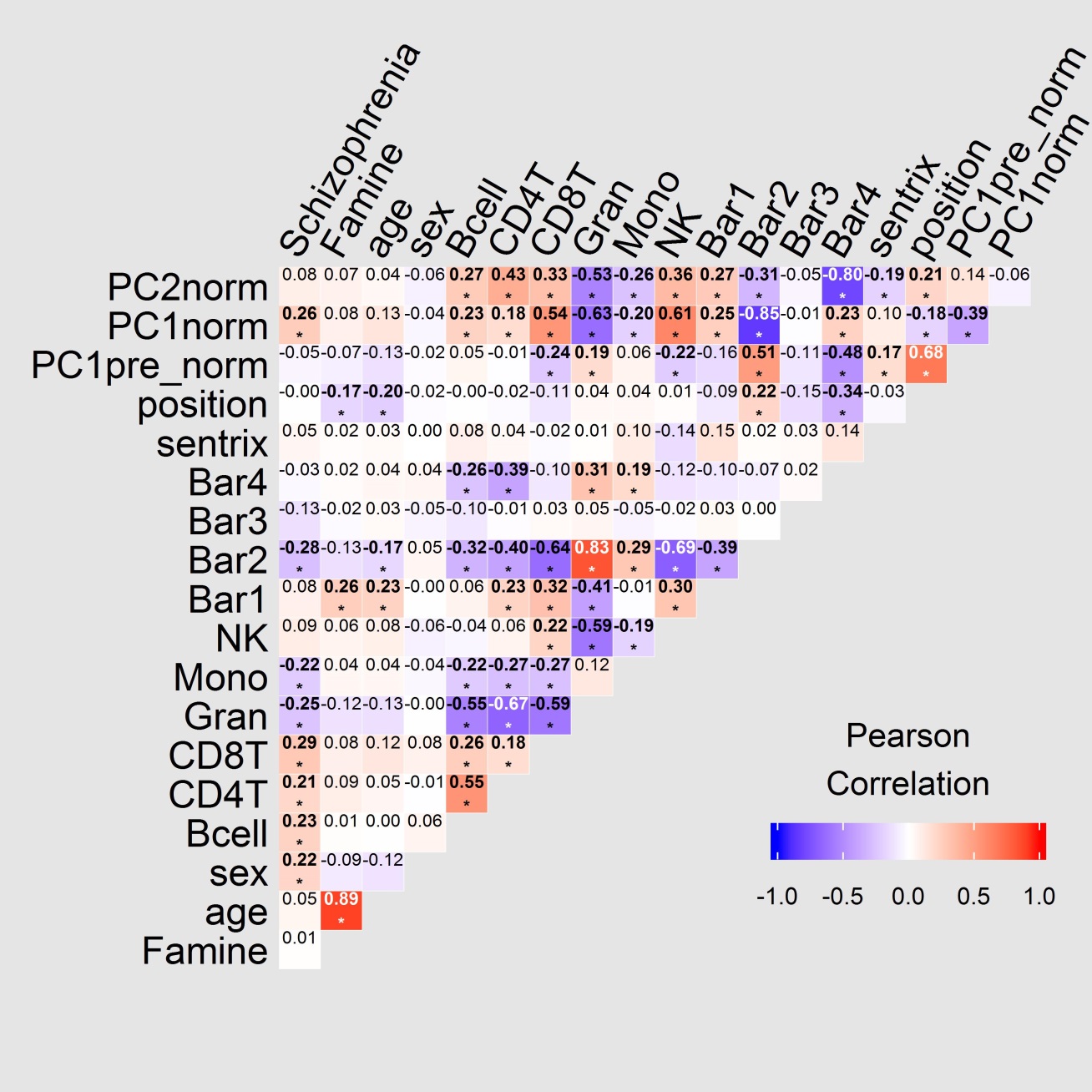


Extended Data Fig. 2 Batch effects, potential confounders and general methylation levels (principal components) before and after application of functional normalization to correct for technical artifacts in the discovery sample(N=153). Correlation between schizophrenia status, maternal famine, methylation (PC) and potential confounders before (3th row from above) and after (top two rows) functional normalization was applied to correct for technical artifacts such as plate and sentrix array. Positive correlations are red and negative correlations are blue the color intensity is related to the size of the correlation coefficient. Significant values are bold and denoted by * p<0.05. Abbreviations: CD8T= CD8 T, CD4T=CD4 T cell, cell NK=Natural Killer, Mono=Monocytes, Gran=Granulocytes, Bar= ancestry estimates calculated according to Barfield et al, sentrix=sentrix array, position=position on sentrix array, col= column on sentrix array, row = row on sentrix array, PC= methylation principal component. The mention of ‘pre’ after the principal component number (e.g. PC1pre_norm), stands for pre functional normalization.


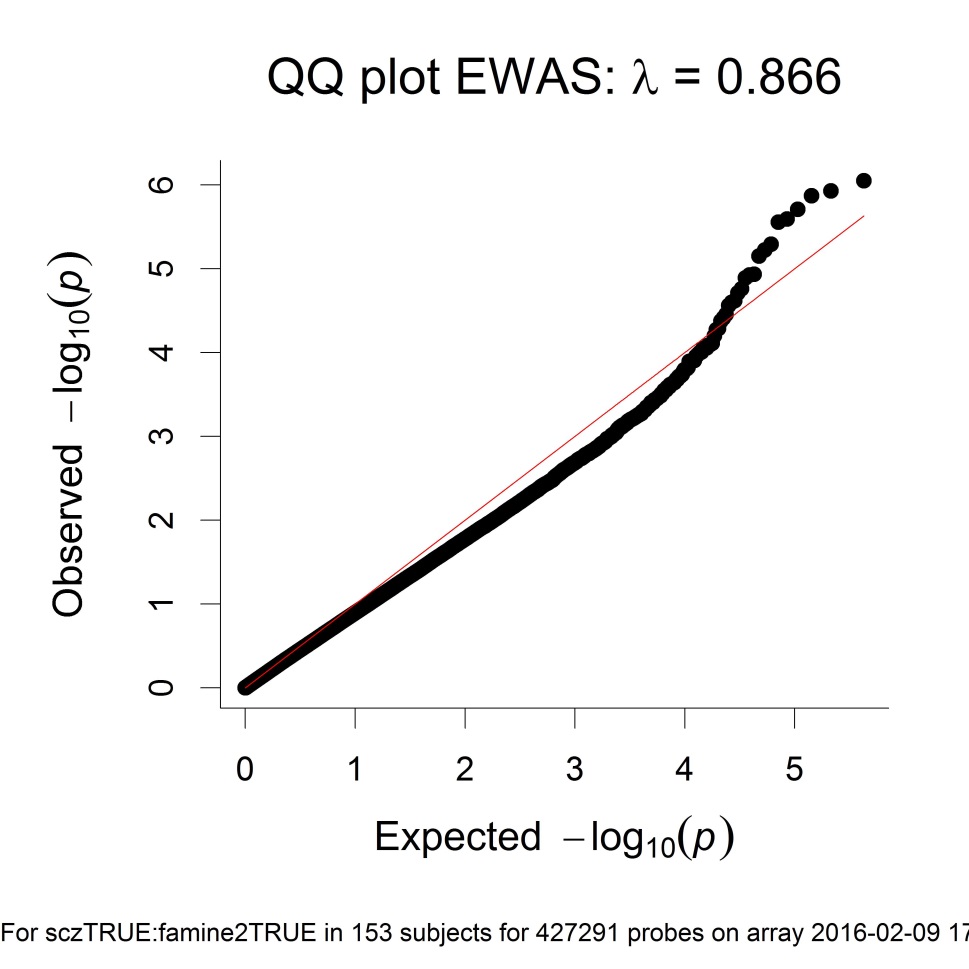


**Extended Data Fig. 3** QQplot of the p-value distribution for the interaction between schizophrenia and famine status in the Chinese famine discovery.

*Genome wide analysis of DNA methylation in the case-control blood samples*

DNA was extracted with the Gentra Puregene Kit (Qiagen, Valencia, CA,USA). After bisulphite conversion (ZYMO Research, Orange, CA, USA) genome-wide DNA methylation levels were obtained using Illumina Infinium HumanMethylation450K BeadChip (Illumina) arrays in a workflow identical to the Chinese famine sample. Intensity read outs, beta- and M-values and cell-type proportion estimates were obtained using the minfi package (version 1.10.2) in Bioconductor^1^. Probes were excluded based on a bead count less than three (n=259 probes) or a detection p-value larger than 0.001 in at least 5% of the samples (n=2,164 probes). Non autosomal and non-specific probes^2^ were discarded as were loci with SNPs of Minor Allele Frequency larger than 1 percent within 1 base pairs of the primer^3^. None of the blood samples had over 1% of failed probes. Successful removal of the sentrix array and position batch effects with functional normalization was confirmed by visual inspection of heatmaps. As expected the cell-type proportion estimates based on the Houseman algorithm^5^ were related to general DNA methylation and therefore added as covariate (see Extended Data Fig. 4). In light of the limited sample size (n=64) none of the individual medication types was added as covariate. The statistical model that was applied genome-wide to identify schizophrenia related DMRs included as indicators a diagnosis of schizophrenia, gender, age and the cell-type estimates (DNA methylation ~ Schizophrenia + age+ gender + CD4T + CD8T + Mono + NK + BCell). We applied the same bump-hunter parameters as in the Chinese famine analysis (500bp window, 95^th^ percentile and 1000 bootstraps) and again only the *DUSP22* region passed the FWER cutoff of 0.05 for schizophrenia as determinant (see Extended data Table 2).

Genotypes for rs12933929 were obtained in samples with sufficient DNA (N=38 controls and N=15 schizophrenia patients) using Fluorescein amidite labeled primers (Forward: CACTTATTAGGTGCCCACTGCCAGC, Reverse-G: GTGAACAGGGTATGTCACCAGGGAGAC, Reverse-A: ACAGGGTATGTCACCAGGGAGAT). Accuracy of the genotyping was confirmed using Sanger sequencing of three samples.


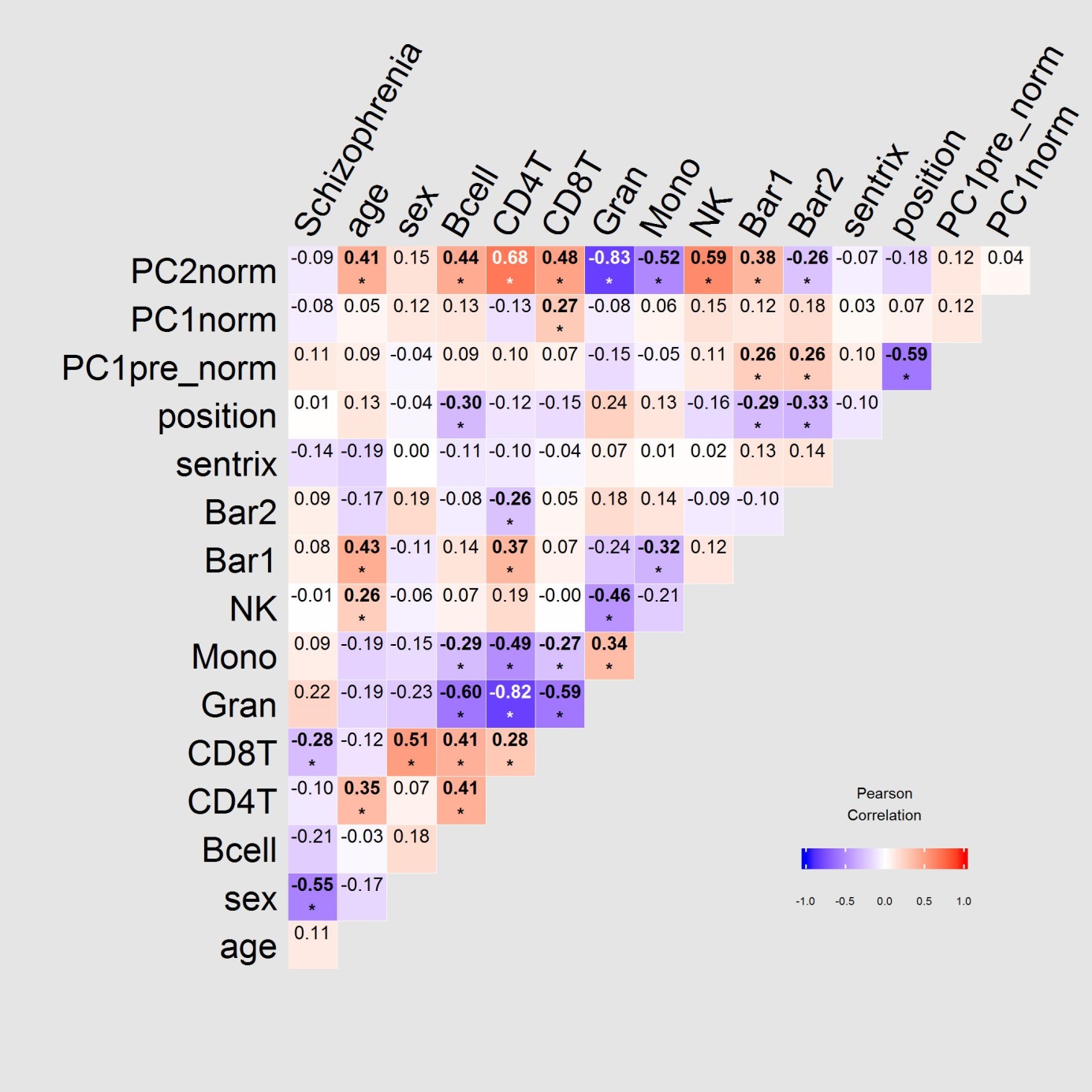


**Extended Data Fig. 4** Batch effects, potential confounders and general methylation levels (principal components) before and after application of functional normalization to correct for technical artifacts in the blood schizophrenia sample (N=64). Correlation between schizophrenia status (scz), methylation(PC) and potential confounders before (3th row from top) and after (top two rows) functional normalization was applied to correct for technical artifacts such as plate and sentrix array. Positive correlations are red and negative correlations are blue, the color intensity is related to the size of the correlation coefficient. Significant values are bold and denoted by * p<0.05. Abbreviations: CD8T= CD8 T, CD4T=CD4 T cell, cell NK=Natural Killer, Mono=Monocytes, Gran=Granulocytes, Bar= ancestry estimates calculated according to Barfield et al, sentrix=sentrix array, position=position on sentrix array, PC= methylation principal component. The mention of ‘pre’ after the principal component number (e.g. PC1pre_norm), stands for pre functional normalization.

*Targeted analysis of DNA methylation in the case-control brain samples*

DNA methylation profiles were quantified using the Infinium Human Methylation 450K BeadChip arrays (for details see ^7^), mRNA expression levels were measured using RNAseq (as previously described^8^) and genotype with either the Illumina Human Hap 650v3,1M Duo V3, or Omni 5M BeadArrays (as previously described ^7^). Sampling quality was checked based on the average intensity and the methylated and unmethylated signals were quantile normalized to correct for type I and II probe design bias using the preprocessQuantile function in the minfi package^1^. To account for technical batch effects the principal components estimated from the microarray negative control background probes (that were strongly associated with processing plate and microarray slide) were added as covariates. In addition, composition estimates for five neuronal cell types in the PFC samples were derived from DNA methylation levels as previously described ^7^. These neuronal cell proportions were related to general DNA methylation and therefore added to the model as covariate. Sex was not associated with diagnosis (p= 0.16), and therefore not included as an adjustment variable. We examined the relationship of DNA methylation as an outcome with diagnosis, controlling for age, race, neuronal cell composition estimates and the first four PCs from the negative control probes (*DUSP22* DNA methylation ~ schizophrenia + age + ethnicity + neuronal cell composition + negControl_PC1 + negControl_PC2 + negControl_PC3 + negControl_PC4).

*Nutritional deprivation of fibroblasts*

Ten fibroblasts cultures were seeded in T25 flasks in growth medium consisting of Dulbecco's Modified Eagle Medium/Nutrient Mixture F-12 with GlutaMAX™ supplement (DMEM/F-12, GlutaMAX™ Supplement, Gibco®), 15% fetal bovine serum (FBS)(Gibco®), 1% Penicillin Streptomycin PenStrep (Gibco®), and in an atmosphere of 95% atmospheric air, 5% CO2, at 37 ˚C. After reaching 70- 80% confluence, the supernatant was removed and the cells were washed three times with phosphate buffered saline (PBS)(BioWhittaker® Reagents, Lonza). Next the cells were cultured in Minimum Essential Medium (MEM) (Gibco®) with or without 15% FBS to mimic famine in vitro. Based on analysis of expression at 24 and 72 hours cells were harvested for analysis of expression (both time points yielded the very similar results). RNA was isolated using TRIzol (TRIzol® Reagent, Ambion®), followed by complementary cDNA synthesis using the Reverse Transcription Kit (QIAGEN). SYBR Green-based Quantitative polymerase chain reaction (qPCR) was performed to quantify mRNA expression level of DUSP22. Ribosomal protein S28 and EF-1alpha were selected as reference genes using the RefFinder program^9^. In addition, mRNA expression levels of the DRD2 gene were measured as an internal reference gene for schizophrenia. The following primers were used: *DUSP22* forward (5’- CATCAGTATCGGCAGTGGCT-3’) and reverse (5’ -GTGCGCCCTTGCTCCTTAT-3’), *DRD2* forward (5’-CAACGGGTCAGACGGGAAG-3’) and reverse (5’- GAATTTCCACTCACCTACCACC-3’), Ribosomal protein S28 forward (5’-GACACGAGCCGATCCATCATC-3’) and reverse (5’-TGACTCCAAAAGGGTGAGCAC-3’), EF-1alpha forward (5’-TGTCGTCATTGGACACGTAGA-3’) and reverse (5’-ACGCTCAGCTTTCAGTTTATCC-3’). Relative mRNA expression was calculated with the Delta Delta CT method with ribosomal protein *S28* and *EF-1alpha* as pooled reference genes by means of their geomean. The response to nutritional deprivation was calculated for each sample as the fold change in gene expression of a sample exposed to nutritional deprivation relative to its unchanged conditions. The differences in *DUSP22* fold changes were analyzed using a paired Wilcoxon rank test for non-parametric within subject comparison of the famine and non-famine condition. DNA was harvested using the QIAamp DNA Mini Kit. DNA was bisulphite converted using ZiMO kits (ZYMO Research, Orange, CA, USA) and genome-wide DNA methylation levels were obtained using Illumina HumanMethylation EPIC BeadChip (Illumina) arrays. Quality control of the methylation data was conducted in a workflow similar to the Chinese famine sample, but adjusted to the newer methylation beadchips. For this purpose we used the developer version of Meffil (https://github.com/perishky/meffil/wiki), the data was generated as part of a larger project that included brain samples. Therefore functional normalization was conducted using control probes of the entire dataset (N=192), but quantile normalization was conducted for the fibroblasts data separately. Outlier detection was set to 5 SD in order to deal with the small sample size. Probes with bead number below 3 in more than 10 percent of the samples were removed (68 CpGs) as were 4639 probes with detection p value larger than 0.01 in more than 10 percent of samples. No samples were removed based on gender mismatch or more than 1 percent of failed probes. Primers for four CpG in *DUSP22* on the 450K array are not present on the EPIC array: cg15383120, cg16103275, cg26220725, cg23208850, of which one cg15383120 is part of the *DUSP22* DMR. We therefore based our analysis on the average methylation of all 31 *DUSP22* CpGs on the EPIC methylation array. Non parametric analysis of average methylation of all *DUSP22* CpGs for the paired observations of all 10 donors for the 72 hours nutritional deprivation compared to the condition with FBS was done using a paired Wilcoxon Signed Rank Test.

*Functional analysis of expression in brain*

To examine group differences in *DUSP22* expression levels, we first excluded all subjects with a RNA Integrity Number (RIN) below eight (excluded schizophrenia N=20; control N= 17). Eight was the lowest RIN value whereby RIN was no longer significantly associated with diagnosis or *DUSP22* expression levels. More lenient analysis with RIN below five yielded very similar results (data not shown). Next, we examined whether group or methylation were related to RNA expression while adjusting for age, the RNA Integrity Number (RIN), the first four PCs of the negative control probes on the methylation microarrays, and neuronal cell composition (RNA expression ~ DNA methylation + schizophrenia + age + gender + Ethnicity + neuronal cell composition + negControl_PC1 + negControl_PC2 + negControl_PC3 + negControl_PC4 + RIN). Finally, the association between RNA expression (as outcome) and DNA methylation was analyzed while adjusting for the genetic background (as covariate) in the linear model (RNA expression ~ DNA methylation + genotype + age+ gender + neuronal cell composition + negControl_PC1 + negControl_PC2 + negControl_PC3 + negControl_PC4+ RIN).

*Functional analysis of expression in blood*

DNA methylation M-values were obtained using the Minfi package^1^ and normalized to correct for type I and II probe design bias using the Beta MIxture Quantile dilation (BMIQ) normalization^10^ as implemented in the watermelon package^11^. Successful batch correction for sentrix array and position with the combat procedure implemented in the sva package^12^ was checked by visual inspection of heatmaps. Since genotype based ancestry principal components one, two and three (PC1-3) as well as the cell-type proportion estimates based on the Houseman algorithm^13^ were related to DNA methylation levels, these were included as covariate in a linear model with DUSP22 expression as outcome and DNA methylation as main determinant (RNA expression ~ DNA methylation + age+ gender + CD4T + CD8T + Mono + NK + BCell + PC1 + PC2 + PC3). Next we examined the association between DNA methylation and RNA expression while controlling for the differences in genetic background by adding these as a covariate to the linear regression model with RNA expression as outcome (RNA expression ~ DNA methylation + age+ gender + CD4T + CD8T + Mono + NK + BCell + PC2 + PC3 + PC4+genotype).

*In situ Hi-C data from human postmortem brain tissue*

Flash frozen postmortem brain tissue was obtained from Human Brain Collection Core (HBCC) of the National Institute of Mental Health (NIMH), US. The anterior cingulate cortex sample used in this study is from a 35-year old unaffected female. Nuclei isolation through extraction, purification, and fluorescence-activated nuclear sorting (FANS) was performed per Kundakovic et al., 2016 ^14^ with the following modification: immediately after douncing, the nuclei are fixed in 1% formaldehyde and rotated at room temperature for 10 minutes. Subsequently, the formaldehyde is quenched with 0.3M glycine. After sorting, approximately 1.5 million NeuN positive nuclei are then subjected to the in situ Hi-C protocol ^15^ without modifications. The HiC library was sequenced on the Illumina 1000 platform (125bp paired-end). The NeuN positive library yielded 170 million reads processed with the HiCPro pipeline^16^ with minimal modification, resulting in 20% chimeric reads. Negative controls included an in situ HiC library made which omitted the crucial ligation step, resulting in 0% chimeric reads. Data are reported in browser-extensible-data-like (BED) format and visualized in the Washington University Epigenome Browser (http://epigenomegateway.wustl.edu/).

**References**

1. Aryee, M. J. *et al.* Minfi: A flexible and comprehensive Bioconductor package for the analysis of Infinium DNA methylation microarrays. *Bioinformatics* **30,** 1363–1369 (2014).

2. Chen, Y. A. *et al.* Discovery of cross-reactive probes and polymorphic CpGs in the Illumina Infinium HumanMethylation450 microarray. *Epigenetics* **8,** 203–209 (2013).

3. Barfield, R. T. *et al.* Accounting for population stratification in DNA methylation studies. *Genet. Epidemiol.* **38,** 231–41 (2014).

4. Fortin, J.-P. *et al.* Functional normalization of 450k methylation array data improves replication in large cancer studies. *Genome Biol.* **15,** 503 (2014).

5. Houseman, E. A. *et al.* DNA methylation arrays as surrogate measures of cell mixture distribution. *BMC.Bioinformatics.* **13,** 86 (2012).

6. Jaffe, A. E. *et al.* Bump hunting to identify differentially methylated regions in epigenetic epidemiology studies. *Int.J.Epidemiol.* **41,** 200–209 (2012).

7. Jaffe, A. E. *et al.* Mapping DNA methylation across development, genotype and schizophrenia in the human frontal cortex. *Nat.Neurosci.* **19,** 40–47 (2016).

8. Punzi, G. *et al.* Increased expression of MARCKS in post-mortem brain of violent suicide completers is related to transcription of a long, noncoding, antisense RNA. *Mol.Psychiatry* (2014).

9. Xie, F., Xiao, P., Chen, D., Xu, L. & Zhang, B. miRDeepFinder: A miRNA analysis tool for deep sequencing of plant small RNAs. *Plant Mol. Biol.* **80,** 75–84 (2012).

10. Teschendorff, A. E. *et al.* A beta-mixture quantile normalization method for correcting probe design bias in Illumina Infinium 450 k DNA methylation data. *Bioinformatics.* **29,** 189–196

11. Schalkwyk, L. C. *et al.* wateRmelon: Illumina 450 methylation array normalization and metrics. R package version 1.4.0. *http://www.bioconductor.org/packages/release/bioc/html/wateRmelon.html* (2013).

12. Leek, J. T., Johnson, W. E., Parker, H. S., Jaffe, A. E. & Storey, J. D. The sva package for removing batch effects and other unwanted variation in high-throughput experiments. *Bioinformatics.* **28,** 882–883

13. Houseman, E. A. *et al.* Model-based clustering of DNA methylation array data: a recursive-partitioning algorithm for high-dimensional data arising as a mixture of beta distributions. *BMC.Bioinformatics.* **9,** 365- (2008).

14. Kundakovic, M. *et al.* Practical Guidelines for High-Resolution Epigenomic Profiling of Nucleosomal Histones in Postmortem Human Brain Tissue. *Biol. Psychiatry* **81,** 162–170 (2017).

15. Rao, U. *et al.* Hippocampal changes associated with early-life adversity and vulnerability to depression. *Biol.Psychiatry* **67,** 357–364

16. Servant, N. *et al.* HiC-Pro: an optimized and flexible pipeline for Hi-C data processing. *Genome Biol.* **16,** 259 (2015).
