# Supplementary material for: Genetic vulnerability to DUSP22 promoter hypermethylation is involved in the relation between in utero famine exposure and schizophrenia
Source: NPJ Schizophr. 2018 Aug 21;4:16. doi: 10.1038/s41537-018-0058-4 (PMC6104043; doi:10.1038/s41537-018-0058-4)
Supplement: Supplementary file 2 — Supplemental table 1 [file 41537_2018_58_MOESM2_ESM.pdf]

snps	cpg	statistic	pvalue	FDR
rs34151874:34989694:G:A	cg07332563	-17.4618	2.33E-52	8.87E-45
rs11646602	cg07332563	-17.4203	3.58E-52	8.87E-45
rs12600198:35112074:G:A	cg07332563	-17.4203	3.58E-52	8.87E-45
rs2173885:34992749:G:A	cg07332563	-17.369	6.10E-52	1.13E-44
rs11646602	cg26668828	-17.2341	2.47E-51	1.64E-44
rs12600198:35112074:G:A	cg26668828	-17.2341	2.47E-51	1.64E-44
rs12445057:34970274:C:G	cg07332563	-17.2294	2.59E-51	1.64E-44
rs12919333:34991336:C:T	cg07332563	-17.2159	2.98E-51	1.64E-44
rs34151874:34989694:G:A	cg26668828	-17.2132	3.07E-51	1.64E-44
rs12933929:35111585:G:A	cg07332563	-17.2126	3.09E-51	1.64E-44
rs11149549:35116314:G:C	cg07332563	-17.2126	3.09E-51	1.64E-44
rs35691226:35132928:T:A	cg07332563	-17.2126	3.09E-51	1.64E-44
rs12596272	cg07332563	-17.2126	3.09E-51	1.64E-44
rs35808007:35156375:G:A	cg07332563	-17.2126	3.09E-51	1.64E-44
rs12447240:34928236:T:C	cg07332563	-17.192	3.82E-51	1.89E-44
rs12929704:34898258:C:A	cg07332563	-17.1318	7.12E-51	3.30E-44
rs1501462:34964515:A:T	cg07332563	-17.0919	1.08E-50	4.70E-44
rs12929704:34898258:C:A	cg26668828	-17.0105	2.50E-50	1.03E-43
rs34006830:34878550:C:A	cg07332563	-16.9969	2.87E-50	1.12E-43
rs12445057:34970274:C:G	cg26668828	-16.9581	4.29E-50	1.59E-43
rs12923277	cg07332563	-16.9383	5.27E-50	1.80E-43
rs12447240:34928236:T:C	cg26668828	-16.9369	5.34E-50	1.80E-43
rs34728702:34896584:A:G	cg07332563	-16.9256	6.00E-50	1.94E-43
rs1433753	cg26668828	-16.8962	8.13E-50	2.52E-43
rs1501462:34964515:A:T	cg26668828	-16.8833	9.29E-50	2.76E-43
rs12444611:35032947:T:G	cg07332563	-16.8626	1.15E-49	3.05E-43
rs34151874:34989694:G:A	cg01171360	-16.8614	1.16E-49	3.05E-43
rs11646602	cg01171360	-16.8566	1.22E-49	3.05E-43
rs12600198:35112074:G:A	cg01171360	-16.8566	1.22E-49	3.05E-43
rs138201488:35007809:G:GT	cg07332563	-16.8558	1.23E-49	3.05E-43
rs2173885:34992749:G:A	cg26668828	-16.7627	3.22E-49	7.49E-43
rs12923277	cg26668828	-16.7625	3.23E-49	7.49E-43
rs1433753	cg07332563	-16.7215	4.92E-49	1.11E-42
rs11645488	cg07332563	-16.7152	5.25E-49	1.15E-42
rs12444879:35211534:T:G	cg07332563	-16.694	6.53E-49	1.21E-42
rs12933929:35111585:G:A	cg26668828	-16.6939	6.54E-49	1.21E-42
rs11149549:35116314:G:C	cg26668828	-16.6939	6.54E-49	1.21E-42
rs35691226:35132928:T:A	cg26668828	-16.6939	6.54E-49	1.21E-42
rs12596272	cg26668828	-16.6939	6.54E-49	1.21E-42
rs35808007:35156375:G:A	cg26668828	-16.6939	6.54E-49	1.21E-42
rs12445057:34970274:C:G	cg01171360	-16.6834	7.28E-49	1.32E-42
rs34151874:34989694:G:A	cg21548813	-16.6762	7.85E-49	1.39E-42
rs2173885:34992749:G:A	cg01171360	-16.6383	1.16E-48	2.00E-42
rs12919333:34991336:C:T	cg01171360	-16.631	1.25E-48	2.11E-42
rs11646602	cg21548813	-16.6215	1.38E-48	2.11E-42
rs12600198:35112074:G:A	cg21548813	-16.6215	1.38E-48	2.11E-42
rs12933929:35111585:G:A	cg01171360	-16.6164	1.45E-48	2.11E-42
rs11149549:35116314:G:C	cg01171360	-16.6164	1.45E-48	2.11E-42
rs35691226:35132928:T:A	cg01171360	-16.6164	1.45E-48	2.11E-42

rs12596272	cg01171360	-16.6164	1.45E-48	2.11E-42
rs35808007:35156375:G:A	cg01171360	-16.6164	1.45E-48	2.11E-42
rs17725554:34809907:A:T	cg26668828	-16.6108	1.54E-48	2.19E-42
rs12447240:34928236:T:C	cg01171360	-16.5947	1.81E-48	2.54E-42
rs12919333:34991336:C:T	cg26668828	-16.536	3.32E-48	4.56E-42
rs17788654:35077080:A:G	cg26668828	-16.5296	3.54E-48	4.78E-42
rs11646005:35076563:C:T	cg07332563	-16.5266	3.65E-48	4.84E-42
rs34151874:34989694:G:A	cg11235426	-16.5239	3.75E-48	4.89E-42
rs12444879:35211534:T:G	cg26668828	-16.5085	4.40E-48	5.63E-42
rs34006830:34878550:C:A	cg26668828	-16.5058	4.52E-48	5.69E-42
rs11645488	cg26668828	-16.5034	4.63E-48	5.74E-42
rs12444611:35032947:T:G	cg26668828	-16.4805	5.86E-48	7.14E-42
rs34728702:34896584:A:G	cg26668828	-16.4734	6.31E-48	7.55E-42
rs1501462:34964515:A:T	cg01171360	-16.4662	6.79E-48	8.01E-42
rs138201488:35007809:G:GT	cg26668828	-16.461	7.16E-48	8.31E-42
rs2173885:34992749:G:A	cg21548813	-16.4476	8.22E-48	9.39E-42
rs12445057:34970274:C:G	cg21548813	-16.444	8.52E-48	9.59E-42
rs12929704:34898258:C:A	cg01171360	-16.4068	1.25E-47	1.38E-41
rs11646602	cg11235426	-16.3562	2.10E-47	2.24E-41
rs12600198:35112074:G:A	cg11235426	-16.3562	2.10E-47	2.24E-41
rs12447240:34928236:T:C	cg21548813	-16.3525	2.18E-47	2.24E-41
rs12929704:34898258:C:A	cg21548813	-16.3518	2.19E-47	2.24E-41
rs12933929:35111585:G:A	cg21548813	-16.3477	2.29E-47	2.24E-41
rs11149549:35116314:G:C	cg21548813	-16.3477	2.29E-47	2.24E-41
rs35691226:35132928:T:A	cg21548813	-16.3477	2.29E-47	2.24E-41
rs12596272	cg21548813	-16.3477	2.29E-47	2.24E-41
rs35808007:35156375:G:A	cg21548813	-16.3477	2.29E-47	2.24E-41
rs34151874:34989694:G:A	cg05064044	-16.3374	2.54E-47	2.45E-41
rs2173885:34992749:G:A	cg11235426	-16.3266	2.84E-47	2.70E-41
rs12923277	cg01171360	-16.3034	3.60E-47	3.38E-41
rs1501462:34964515:A:T	cg21548813	-16.2802	4.56E-47	4.24E-41
rs1973278:34856488:G:A	cg26668828	-16.2732	4.91E-47	4.50E-41
rs17788654:35077080:A:G	cg07332563	-16.2681	5.17E-47	4.68E-41
rs11646602	cg05064044	-16.2534	6.01E-47	5.29E-41
rs12600198:35112074:G:A	cg05064044	-16.2534	6.01E-47	5.29E-41
rs2163975:34884821:C:G	cg07332563	-16.2526	6.05E-47	5.29E-41
rs34006830:34878550:C:A	cg01171360	-16.2502	6.21E-47	5.36E-41
rs12919333:34991336:C:T	cg21548813	-16.2407	6.84E-47	5.84E-41
rs12444879:35211534:T:G	cg01171360	-16.2381	7.03E-47	5.93E-41
rs1973278:34856488:G:A	cg07332563	-16.233	7.40E-47	6.17E-41
rs12444611:35032947:T:G	cg01171360	-16.2128	9.10E-47	7.51E-41
rs2163975:34884821:C:G	cg26668828	-16.2074	9.62E-47	7.85E-41
rs12933929:35111585:G:A	cg11235426	-16.1961	1.08E-46	8.35E-41
rs11149549:35116314:G:C	cg11235426	-16.1961	1.08E-46	8.35E-41
rs35691226:35132928:T:A	cg11235426	-16.1961	1.08E-46	8.35E-41
rs12596272	cg11235426	-16.1961	1.08E-46	8.35E-41
rs35808007:35156375:G:A	cg11235426	-16.1961	1.08E-46	8.35E-41
rs12919333:34991336:C:T	cg11235426	-16.1916	1.13E-46	8.65E-41
rs11648847:35257891:A:T	cg07332563	-16.1906	1.14E-46	8.65E-41
rs11646005:35076563:C:T	cg26668828	-16.179	1.29E-46	9.64E-41

rs76846224:35065215:A:G	cg07332563	-16.1769	1.31E-46	9.75E-41
rs34728702:34896584:A:G	cg01171360	-16.1718	1.38E-46	1.02E-40
rs12923277	cg21548813	-16.1532	1.67E-46	1.22E-40
rs138201488:35007809:G:GT	cg01171360	-16.1286	2.15E-46	1.55E-40
rs12929704:34898258:C:A	cg11235426	-16.1278	2.17E-46	1.55E-40
rs34006830:34878550:C:A	cg21548813	-16.1205	2.34E-46	1.64E-40
rs12447240:34928236:T:C	cg05064044	-16.1199	2.35E-46	1.64E-40
rs12445057:34970274:C:G	cg11235426	-16.119	2.37E-46	1.64E-40
rs12445057:34970274:C:G	cg05064044	-16.0987	2.92E-46	2.00E-40
rs12447240:34928236:T:C	cg11235426	-16.0877	3.26E-46	2.22E-40
rs34728702:34896584:A:G	cg21548813	-16.0802	3.52E-46	2.38E-40
rs2173885:34992749:G:A	cg05064044	-16.0749	3.72E-46	2.49E-40
rs11646005:35076563:C:T	cg01171360	-16.0632	4.19E-46	2.78E-40
rs1433753	cg21548813	-16.049	4.84E-46	3.18E-40
rs1433753	cg01171360	-16.0268	6.07E-46	3.96E-40
rs1501462:34964515:A:T	cg05064044	-16.0245	6.21E-46	4.01E-40
rs12929704:34898258:C:A	cg05064044	-16.0156	6.81E-46	4.36E-40
rs34006830:34878550:C:A	cg11235426	-16.0048	7.60E-46	4.82E-40
rs1501462:34964515:A:T	cg11235426	-15.9998	8.00E-46	4.93E-40
rs12933929:35111585:G:A	cg05064044	-15.9977	8.17E-46	4.93E-40
rs11149549:35116314:G:C	cg05064044	-15.9977	8.17E-46	4.93E-40
rs35691226:35132928:T:A	cg05064044	-15.9977	8.17E-46	4.93E-40
rs12596272	cg05064044	-15.9977	8.17E-46	4.93E-40
rs35808007:35156375:G:A	cg05064044	-15.9977	8.17E-46	4.93E-40
rs34728702:34896584:A:G	cg11235426	-15.9685	1.10E-45	6.59E-40
rs12919333:34991336:C:T	cg05064044	-15.9642	1.15E-45	6.83E-40
rs11645488	cg01171360	-15.9623	1.17E-45	6.91E-40
rs12444879:35211534:T:G	cg21548813	-15.9282	1.66E-45	9.70E-40
rs1973278:34856488:G:A	cg01171360	-15.9265	1.69E-45	9.79E-40
rs12923277	cg11235426	-15.9238	1.73E-45	9.98E-40
rs2163975:34884821:C:G	cg01171360	-15.9118	1.96E-45	1.12E-39
rs2163977	cg07332563	-15.9087	2.02E-45	1.14E-39
rs11648847:35257891:A:T	cg26668828	-15.9084	2.03E-45	1.14E-39
rs11648847:35257891:A:T	cg01171360	-15.8777	2.77E-45	1.55E-39
rs2163977	cg26668828	-15.866	3.12E-45	1.73E-39
rs12444611:35032947:T:G	cg21548813	-15.8183	5.07E-45	2.79E-39
rs76846224:35065215:A:G	cg26668828	-15.817	5.14E-45	2.80E-39
rs17725554:34809907:A:T	cg07332563	-15.8151	5.23E-45	2.84E-39
rs1433753	cg11235426	-15.8106	5.48E-45	2.95E-39
rs11646005:35076563:C:T	cg21548813	-15.8012	6.03E-45	3.22E-39
rs34006830:34878550:C:A	cg05064044	-15.7972	6.28E-45	3.33E-39
rs138201488:35007809:G:GT	cg21548813	-15.7957	6.37E-45	3.36E-39
rs12923277	cg05064044	-15.782	7.32E-45	3.83E-39
rs34728702:34896584:A:G	cg05064044	-15.7614	9.03E-45	4.69E-39
rs12444879:35211534:T:G	cg11235426	-15.7513	1.00E-44	5.16E-39
rs17725554:34809907:A:T	cg01171360	-15.7483	1.03E-44	5.28E-39
rs11645488	cg21548813	-15.7472	1.04E-44	5.29E-39
rs17788654:35077080:A:G	cg01171360	-15.7467	1.05E-44	5.29E-39
rs12149595:35259917:G:A	cg26668828	-15.7346	1.19E-44	5.95E-39
rs1433753	cg05064044	-15.7236	1.32E-44	6.60E-39

rs1973278:34856488:G:A	cg21548813	-15.7128	1.48E-44	7.32E-39
rs2163975:34884821:C:G	cg21548813	-15.6816	2.03E-44	9.97E-39
rs17788654:35077080:A:G	cg21548813	-15.659	2.55E-44	1.25E-38
rs12444611:35032947:T:G	cg11235426	-15.6563	2.62E-44	1.27E-38
rs138201488:35007809:G:GT	cg11235426	-15.6501	2.79E-44	1.35E-38
rs11646005:35076563:C:T	cg11235426	-15.6366	3.20E-44	1.53E-38
rs2163977	cg01171360	-15.6236	3.65E-44	1.74E-38
rs1973278:34856488:G:A	cg11235426	-15.6091	4.23E-44	2.00E-38
rs2163975:34884821:C:G	cg11235426	-15.5792	5.72E-44	2.69E-38
rs76846224:35065215:A:G	cg01171360	-15.5785	5.76E-44	2.69E-38
rs12444879:35211534:T:G	cg05064044	-15.5748	5.98E-44	2.78E-38
rs11645488	cg11235426	-15.5056	1.20E-43	5.55E-38
rs11646005:35076563:C:T	cg05064044	-15.4881	1.43E-43	6.58E-38
rs17725554:34809907:A:T	cg21548813	-15.4449	2.22E-43	1.01E-37
rs12149595:35259917:G:A	cg07332563	-15.4434	2.25E-43	1.02E-37
rs11646602	cg03395511	-15.4303	2.57E-43	1.15E-37
rs12600198:35112074:G:A	cg03395511	-15.4303	2.57E-43	1.15E-37
rs11645488	cg05064044	-15.4244	2.73E-43	1.21E-37
rs34151874:34989694:G:A	cg03395511	-15.4229	2.77E-43	1.22E-37
rs11648847:35257891:A:T	cg21548813	-15.415	3.00E-43	1.32E-37
rs12444611:35032947:T:G	cg05064044	-15.4093	3.18E-43	1.39E-37
rs1973278:34856488:G:A	cg05064044	-15.3975	3.58E-43	1.56E-37
rs138201488:35007809:G:GT	cg05064044	-15.3866	4.00E-43	1.73E-37
rs17788654:35077080:A:G	cg11235426	-15.3688	4.78E-43	2.05E-37
rs17788654:35077080:A:G	cg05064044	-15.3673	4.85E-43	2.07E-37
rs2173885:34992749:G:A	cg03395511	-15.3668	4.88E-43	2.07E-37
rs2163977	cg21548813	-15.3664	4.90E-43	2.07E-37
rs2163975:34884821:C:G	cg05064044	-15.3574	5.36E-43	2.25E-37
rs12445057:34970274:C:G	cg03395511	-15.3001	9.55E-43	3.98E-37
rs12919333:34991336:C:T	cg03395511	-15.2806	1.16E-42	4.82E-37
rs12933929:35111585:G:A	cg03395511	-15.2613	1.41E-42	5.69E-37
rs11149549:35116314:G:C	cg03395511	-15.2613	1.41E-42	5.69E-37
rs35691226:35132928:T:A	cg03395511	-15.2613	1.41E-42	5.69E-37
rs12596272	cg03395511	-15.2613	1.41E-42	5.69E-37
rs35808007:35156375:G:A	cg03395511	-15.2613	1.41E-42	5.69E-37
rs11648847:35257891:A:T	cg05064044	-15.2583	1.45E-42	5.83E-37
rs2163977	cg11235426	-15.2364	1.81E-42	7.23E-37
rs12447240:34928236:T:C	cg03395511	-15.2257	2.02E-42	8.01E-37
rs17725554:34809907:A:T	cg11235426	-15.2023	2.55E-42	1.01E-36
rs11648847:35257891:A:T	cg11235426	-15.1934	2.79E-42	1.10E-36
rs12926980	cg26668828	-15.1912	2.85E-42	1.12E-36
rs17725554:34809907:A:T	cg05064044	-15.1895	2.90E-42	1.13E-36
rs11644352:34724788:G:T	cg26668828	-15.1834	3.08E-42	1.19E-36
rs1501462:34964515:A:T	cg03395511	-15.157	4.02E-42	1.55E-36
rs12929704:34898258:C:A	cg03395511	-15.147	4.44E-42	1.70E-36
rs76846224:35065215:A:G	cg21548813	-15.0982	7.25E-42	2.76E-36
rs2163977	cg05064044	-15.076	9.06E-42	3.43E-36
rs11647994	cg07332563	-15.0539	1.13E-41	4.26E-36
rs34006830:34878550:C:A	cg03395511	-15.0342	1.38E-41	5.16E-36
rs12149595:35259917:G:A	cg01171360	-15.033	1.39E-41	5.20E-36

rs12926980	cg07332563	-14.9985	1.97E-41	7.30E-36
rs34728702:34896584:A:G	cg03395511	-14.9792	2.38E-41	8.81E-36
rs76846224:35065215:A:G	cg11235426	-14.9558	3.01E-41	1.11E-35
rs34769185:34654349:G:A	cg26668828	-14.9554	3.03E-41	1.11E-35
rs1433753	cg03395511	-14.941	3.49E-41	1.27E-35
rs12923277	cg03395511	-14.9369	3.64E-41	1.32E-35
rs12926980	cg01171360	-14.901	5.21E-41	1.88E-35
rs12444879:35211534:T:G	cg03395511	-14.8858	6.06E-41	2.17E-35
rs11647994	cg26668828	-14.8686	7.19E-41	2.57E-35
rs12444611:35032947:T:G	cg03395511	-14.7808	1.73E-40	6.13E-35
rs11646005:35076563:C:T	cg03395511	-14.78	1.74E-40	6.15E-35
rs138201488:35007809:G:GT	cg03395511	-14.7529	2.28E-40	8.01E-35
rs76846224:35065215:A:G	cg05064044	-14.7329	2.78E-40	9.73E-35
rs12149595:35259917:G:A	cg21548813	-14.7205	3.14E-40	1.10E-34
rs12599106:34498025:T:A	cg26668828	-14.7164	3.27E-40	1.14E-34
rs71378663:34532030:T:C	cg26668828	-14.6852	4.46E-40	1.54E-34
rs17788654:35077080:A:G	cg03395511	-14.6681	5.29E-40	1.82E-34
rs11645488	cg03395511	-14.6537	6.10E-40	2.09E-34
rs11647994	cg01171360	-14.633	7.49E-40	2.55E-34
rs1973278:34856488:G:A	cg03395511	-14.6249	8.12E-40	2.75E-34
rs12149595:35259917:G:A	cg05064044	-14.6096	9.44E-40	3.19E-34
rs2163975:34884821:C:G	cg03395511	-14.5984	1.06E-39	3.55E-34
rs34151874:34989694:G:A	cg18110333	-14.5896	1.15E-39	3.85E-34
rs11644352:34724788:G:T	cg07332563	-14.5831	1.23E-39	4.09E-34
rs11646602	cg18110333	-14.5737	1.35E-39	4.45E-34
rs12600198:35112074:G:A	cg18110333	-14.5737	1.35E-39	4.45E-34
rs2004779:34631340:T:C	cg26668828	-14.5518	1.68E-39	5.50E-34
rs7204085:34522955:C:T	cg26668828	-14.5168	2.37E-39	7.74E-34
rs1433753	cg18110333	-14.5146	2.42E-39	7.88E-34
rs56403495:34450108:A:T	cg26668828	-14.4843	3.27E-39	1.05E-33
rs199982235:34463368:T:C	cg26668828	-14.4843	3.27E-39	1.05E-33
rs11644352:34724788:G:T	cg01171360	-14.4739	3.62E-39	1.16E-33
rs34922677:34460309:C:T	cg26668828	-14.4641	3.99E-39	1.27E-33
rs140754489:34461167:G:A	cg26668828	-14.4641	3.99E-39	1.27E-33
rs12445410:34520436:A:C	cg26668828	-14.455	4.36E-39	1.38E-33
rs113572057:34190042:G:T	cg26668828	-14.4515	4.51E-39	1.43E-33
rs11648847:35257891:A:T	cg03395511	-14.436	5.26E-39	1.66E-33
rs10438601:34625895:C:T	cg26668828	-14.3995	7.54E-39	2.36E-33
rs12149595:35259917:G:A	cg11235426	-14.3988	7.59E-39	2.37E-33
rs35925518:34534674:A:G	cg26668828	-14.3931	8.03E-39	2.50E-33
rs17725554:34809907:A:T	cg03395511	-14.3921	8.11E-39	2.51E-33
rs2173885:34992749:G:A	cg18110333	-14.3576	1.14E-38	3.51E-33
rs12445057:34970274:C:G	cg18110333	-14.3494	1.24E-38	3.79E-33
rs12933929:35111585:G:A	cg18110333	-14.311	1.80E-38	5.42E-33
rs11149549:35116314:G:C	cg18110333	-14.311	1.80E-38	5.42E-33
rs35691226:35132928:T:A	cg18110333	-14.311	1.80E-38	5.42E-33
rs12596272	cg18110333	-14.311	1.80E-38	5.42E-33
rs35808007:35156375:G:A	cg18110333	-14.311	1.80E-38	5.42E-33
rs12926980	cg21548813	-14.3095	1.83E-38	5.48E-33
rs12447240:34928236:T:C	cg18110333	-14.3032	1.95E-38	5.81E-33

rs2163977	cg03395511	-14.2987	2.04E-38	6.05E-33
rs17788654:35077080:A:G	cg18110333	-14.2933	2.15E-38	6.35E-33
rs12929704:34898258:C:A	cg18110333	-14.2905	2.21E-38	6.50E-33
rs11647994	cg21548813	-14.2703	2.69E-38	7.90E-33
rs11647994	cg05064044	-14.2603	2.97E-38	8.68E-33
rs34151874:34989694:G:A	cg15383120	-14.2568	3.07E-38	8.94E-33
rs11646602	cg15383120	-14.255	3.13E-38	9.04E-33
rs12600198:35112074:G:A	cg15383120	-14.255	3.13E-38	9.04E-33
rs12926980	cg11235426	-14.2417	3.56E-38	1.03E-32
rs2173885:34992749:G:A	cg15383120	-14.239	3.66E-38	1.05E-32
rs12444879:35211534:T:G	cg18110333	-14.2346	3.82E-38	1.09E-32
rs1501462:34964515:A:T	cg18110333	-14.2287	4.05E-38	1.15E-32
rs35721740:34708670:G:A	cg26668828	-14.2258	4.17E-38	1.18E-32
rs76039834:34598453:G:A	cg26668828	-14.2037	5.18E-38	1.46E-32
rs34769185:34654349:G:A	cg07332563	-14.2017	5.28E-38	1.49E-32
rs12919333:34991336:C:T	cg15383120	-14.1975	5.50E-38	1.54E-32
rs12919333:34991336:C:T	cg18110333	-14.1941	5.68E-38	1.59E-32
rs17723574:34602945:C:G	cg26668828	-14.1474	8.99E-38	2.50E-32
rs12599106:34498025:T:A	cg07332563	-14.1455	9.16E-38	2.54E-32
rs12926980	cg05064044	-14.1449	9.21E-38	2.54E-32
rs76846224:35065215:A:G	cg03395511	-14.1232	1.14E-37	3.13E-32
rs11647994	cg11235426	-14.117	1.21E-37	3.32E-32
rs12445057:34970274:C:G	cg15383120	-14.1148	1.24E-37	3.38E-32
rs12933929:35111585:G:A	cg15383120	-14.112	1.27E-37	3.41E-32
rs11149549:35116314:G:C	cg15383120	-14.112	1.27E-37	3.41E-32
rs35691226:35132928:T:A	cg15383120	-14.112	1.27E-37	3.41E-32
rs12596272	cg15383120	-14.112	1.27E-37	3.41E-32
rs35808007:35156375:G:A	cg15383120	-14.112	1.27E-37	3.41E-32
rs34769185:34654349:G:A	cg01171360	-14.0937	1.52E-37	4.06E-32
rs12923277	cg18110333	-14.0874	1.62E-37	4.31E-32
rs11644352:34724788:G:T	cg21548813	-14.0862	1.64E-37	4.34E-32
rs12599106:34498025:T:A	cg01171360	-14.08	1.74E-37	4.60E-32
rs17725554:34809907:A:T	cg18110333	-14.0613	2.09E-37	5.50E-32
rs11645488	cg18110333	-14.0547	2.23E-37	5.84E-32
rs34006830:34878550:C:A	cg18110333	-14.0499	2.33E-37	6.10E-32
rs34728702:34896584:A:G	cg18110333	-14.03	2.84E-37	7.39E-32
rs12447240:34928236:T:C	cg15383120	-14.0279	2.90E-37	7.52E-32
rs12597352:34638507:A:T	cg26668828	-14.0141	3.31E-37	8.57E-32
rs12929704:34898258:C:A	cg15383120	-14.0055	3.60E-37	9.29E-32
rs8056602:34693165:G:T	cg26668828	-13.9947	4.00E-37	1.03E-31
rs11646005:35076563:C:T	cg18110333	-13.9747	4.86E-37	1.25E-31
rs56403495:34450108:A:T	cg07332563	-13.9725	4.97E-37	1.26E-31
rs199982235:34463368:T:C	cg07332563	-13.9725	4.97E-37	1.26E-31
rs7204085:34522955:C:T	cg07332563	-13.9697	5.11E-37	1.30E-31
rs1501462:34964515:A:T	cg15383120	-13.9622	5.49E-37	1.39E-31
rs12921999:34197985:G:C	cg26668828	-13.9578	5.74E-37	1.43E-31
rs66873518:34208117:C:T	cg26668828	-13.9578	5.74E-37	1.43E-31
rs17782957:34274796:T:C	cg26668828	-13.9578	5.74E-37	1.43E-31
rs10083785:34279993:A:G	cg26668828	-13.9578	5.74E-37	1.43E-31
rs71378663:34532030:T:C	cg01171360	-13.9526	6.04E-37	1.50E-31

rs71378663:34532030:T:C	cg07332563	-13.9482	6.30E-37	1.56E-31
rs11644352:34724788:G:T	cg11235426	-13.9425	6.66E-37	1.64E-31
rs12444611:35032947:T:G	cg18110333	-13.9374	7.00E-37	1.72E-31
rs12933921:34400371:G:C	cg26668828	-13.9271	7.74E-37	1.90E-31
rs55701516:34192380:T:C	cg26668828	-13.9207	8.24E-37	2.01E-31
rs12445410:34520436:A:C	cg07332563	-13.9155	8.66E-37	2.11E-31
rs138201488:35007809:G:GT	cg18110333	-13.9078	9.34E-37	2.27E-31
rs34006830:34878550:C:A	cg15383120	-13.9049	9.61E-37	2.32E-31
rs35721740:34708670:G:A	cg07332563	-13.8872	1.14E-36	2.75E-31
rs7204085:34522955:C:T	cg01171360	-13.872	1.32E-36	3.17E-31
rs11644352:34724788:G:T	cg05064044	-13.872	1.32E-36	3.17E-31
rs12445410:34520436:A:C	cg01171360	-13.8712	1.33E-36	3.18E-31
rs34769185:34654349:G:A	cg21548813	-13.863	1.44E-36	3.41E-31
rs34728702:34896584:A:G	cg15383120	-13.8629	1.45E-36	3.41E-31
rs56403495:34450108:A:T	cg01171360	-13.8628	1.45E-36	3.41E-31
rs199982235:34463368:T:C	cg01171360	-13.8628	1.45E-36	3.41E-31
rs3853177:34498532:A:G	cg26668828	-13.8416	1.78E-36	4.18E-31
rs12923277	cg15383120	-13.8339	1.92E-36	4.49E-31
rs2004779:34631340:T:C	cg01171360	-13.8188	2.22E-36	5.18E-31
rs1433753	cg15383120	-13.817	2.26E-36	5.26E-31
rs11648801	cg26668828	-13.7966	2.75E-36	6.39E-31
rs11648847:35257891:A:T	cg18110333	-13.7946	2.81E-36	6.49E-31
rs113572057:34190042:G:T	cg07332563	-13.7944	2.81E-36	6.49E-31
rs2004779:34631340:T:C	cg07332563	-13.7926	2.86E-36	6.58E-31
rs12444611:35032947:T:G	cg15383120	-13.7893	2.95E-36	6.77E-31
rs34922677:34460309:C:T	cg07332563	-13.7839	3.11E-36	7.09E-31
rs140754489:34461167:G:A	cg07332563	-13.7839	3.11E-36	7.09E-31
rs138201488:35007809:G:GT	cg15383120	-13.7619	3.85E-36	8.75E-31
rs12149595:35259917:G:A	cg03395511	-13.7577	4.01E-36	9.09E-31
rs12444879:35211534:T:G	cg15383120	-13.7485	4.39E-36	9.91E-31
rs34922677:34460309:C:T	cg01171360	-13.7473	4.44E-36	9.96E-31
rs140754489:34461167:G:A	cg01171360	-13.7473	4.44E-36	9.96E-31
rs1973278:34856488:G:A	cg18110333	-13.7445	4.56E-36	1.02E-30
rs35721740:34708670:G:A	cg01171360	-13.728	5.35E-36	1.19E-30
rs8056602:34693165:G:T	cg07332563	-13.7267	5.42E-36	1.20E-30
rs8056602:34693165:G:T	cg01171360	-13.7215	5.70E-36	1.26E-30
rs1391904:34548934:C:T	cg26668828	-13.6994	7.06E-36	1.56E-30
rs2163975:34884821:C:G	cg18110333	-13.6943	7.42E-36	1.64E-30
rs113572057:34190042:G:T	cg01171360	-13.6927	7.53E-36	1.66E-30
rs35925518:34534674:A:G	cg07332563	-13.6866	7.99E-36	1.75E-30
rs34769185:34654349:G:A	cg05064044	-13.67	9.39E-36	2.05E-30
rs11645488	cg15383120	-13.6698	9.41E-36	2.05E-30
rs34584967:34475725:T:G	cg26668828	-13.6665	9.70E-36	2.11E-30
rs35925518:34534674:A:G	cg01171360	-13.6629	1.01E-35	2.18E-30
rs8047974	cg26668828	-13.6571	1.06E-35	2.30E-30
rs12931636	cg26668828	-13.6525	1.11E-35	2.39E-30
rs12599106:34498025:T:A	cg21548813	-13.6475	1.17E-35	2.50E-30
rs11646005:35076563:C:T	cg15383120	-13.6201	1.52E-35	3.25E-30
rs34769185:34654349:G:A	cg11235426	-13.6008	1.83E-35	3.91E-30
rs10438601:34625895:C:T	cg07332563	-13.5981	1.88E-35	4.00E-30

rs35080788:34192617:G:A	cg26668828	-13.5902	2.03E-35	4.31E-30
rs34151874:34989694:G:A	cg01516881	-13.5705	2.45E-35	5.19E-30
rs71378081:34345999:TAAAAATAAACA:⁻	cg26668828	-13.5536	2.89E-35	6.07E-30
rs11640596:34347277:T:C	cg26668828	-13.5536	2.89E-35	6.07E-30
rs12149595:35259917:G:A	cg18110333	-13.5519	2.93E-35	6.16E-30
rs71378663:34532030:T:C	cg21548813	-13.5472	3.07E-35	6.42E-30
rs12445057:34970274:C:G	cg01516881	-13.544	3.17E-35	6.61E-30
rs1973278:34856488:G:A	cg15383120	-13.5391	3.32E-35	6.91E-30
rs10438601:34625895:C:T	cg01171360	-13.5328	3.53E-35	7.32E-30
rs2163975:34884821:C:G	cg15383120	-13.5122	4.30E-35	8.90E-30
rs17788654:35077080:A:G	cg15383120	-13.4669	6.65E-35	1.37E-29
rs7204085:34522955:C:T	cg21548813	-13.4495	7.86E-35	1.62E-29
rs12447240:34928236:T:C	cg01516881	-13.4387	8.72E-35	1.79E-29
rs2163977	cg18110333	-13.4359	8.96E-35	1.83E-29
rs2004779:34631340:T:C	cg21548813	-13.4358	8.97E-35	1.83E-29
rs2173885:34992749:G:A	cg01516881	-13.4284	9.63E-35	1.96E-29
rs56403495:34450108:A:T	cg21548813	-13.4278	9.69E-35	1.96E-29
rs199982235:34463368:T:C	cg21548813	-13.4278	9.69E-35	1.96E-29
rs12933921:34400371:G:C	cg01171360	-13.4236	1.01E-34	2.03E-29
rs12599106:34498025:T:A	cg11235426	-13.4144	1.10E-34	2.22E-29
rs11647994	cg03395511	-13.4109	1.14E-34	2.27E-29
rs11646602	cg01516881	-13.4109	1.14E-34	2.27E-29
rs12600198:35112074:G:A	cg01516881	-13.4109	1.14E-34	2.27E-29
rs8054095:34309883:T:C	cg26668828	-13.4061	1.19E-34	2.37E-29
rs76039834:34598453:G:A	cg01171360	-13.3898	1.39E-34	2.77E-29
rs76039834:34598453:G:A	cg07332563	-13.3783	1.56E-34	3.08E-29
rs1501462:34964515:A:T	cg01516881	-13.3619	1.82E-34	3.60E-29
rs71378663:34532030:T:C	cg11235426	-13.3578	1.89E-34	3.73E-29
rs12599106:34498025:T:A	cg05064044	-13.3528	1.99E-34	3.91E-29
rs12445410:34520436:A:C	cg21548813	-13.347	2.10E-34	4.12E-29
rs76846224:35065215:A:G	cg18110333	-13.3413	2.22E-34	4.32E-29
rs12926980	cg03395511	-13.3411	2.22E-34	4.32E-29
rs34922677:34460309:C:T	cg21548813	-13.3409	2.23E-34	4.32E-29
rs140754489:34461167:G:A	cg21548813	-13.3409	2.23E-34	4.32E-29
rs2004779:34631340:T:C	cg05064044	-13.3356	2.34E-34	4.53E-29
rs1391904:34548934:C:T	cg01171360	-13.3165	2.81E-34	5.43E-29
rs17723574:34602945:C:G	cg07332563	-13.3057	3.12E-34	6.00E-29
rs17723574:34602945:C:G	cg01171360	-13.305	3.14E-34	6.03E-29
rs12933929:35111585:G:A	cg01516881	-13.3029	3.20E-34	6.07E-29
rs11149549:35116314:G:C	cg01516881	-13.3029	3.20E-34	6.07E-29
rs35691226:35132928:T:A	cg01516881	-13.3029	3.20E-34	6.07E-29
rs12596272	cg01516881	-13.3029	3.20E-34	6.07E-29
rs35808007:35156375:G:A	cg01516881	-13.3029	3.20E-34	6.07E-29
rs12933921:34400371:G:C	cg07332563	-13.2938	3.50E-34	6.61E-29
rs113572057:34190042:G:T	cg21548813	-13.2828	3.88E-34	7.32E-29
rs35721740:34708670:G:A	cg21548813	-13.2736	4.24E-34	7.98E-29
rs1391904:34548934:C:T	cg07332563	-13.2716	4.32E-34	8.11E-29
rs55701516:34192380:T:C	cg07332563	-13.27	4.39E-34	8.21E-29
rs76846224:35065215:A:G	cg15383120	-13.263	4.69E-34	8.76E-29
rs12919333:34991336:C:T	cg01516881	-13.262	4.74E-34	8.82E-29

rs12929704:34898258:C:A	cg01516881	-13.2565	5.00E-34	9.28E-29
rs35925518:34534674:A:G	cg21548813	-13.2533	5.15E-34	9.54E-29
rs71378663:34532030:T:C	cg05064044	-13.2424	5.71E-34	1.06E-28
rs11648847:35257891:A:T	cg15383120	-13.2415	5.76E-34	1.06E-28
rs12597352:34638507:A:T	cg01171360	-13.2349	6.14E-34	1.13E-28
rs10438601:34625895:C:T	cg21548813	-13.2295	6.47E-34	1.19E-28
rs2004779:34631340:T:C	cg11235426	-13.2284	6.53E-34	1.19E-28
rs7204085:34522955:C:T	cg11235426	-13.2283	6.54E-34	1.19E-28
rs2163977	cg15383120	-13.2281	6.55E-34	1.19E-28
rs56403495:34450108:A:T	cg11235426	-13.219	7.15E-34	1.29E-28
rs199982235:34463368:T:C	cg11235426	-13.219	7.15E-34	1.29E-28
rs3853177:34498532:A:G	cg07332563	-13.2098	7.80E-34	1.41E-28
rs12921999:34197985:G:C	cg07332563	-13.2057	8.11E-34	1.45E-28
rs66873518:34208117:C:T	cg07332563	-13.2057	8.11E-34	1.45E-28
rs17782957:34274796:T:C	cg07332563	-13.2057	8.11E-34	1.45E-28
rs10083785:34279993:A:G	cg07332563	-13.2057	8.11E-34	1.45E-28
rs11648801	cg07332563	-13.1992	8.63E-34	1.54E-28
rs3853177:34498532:A:G	cg01171360	-13.1841	9.97E-34	1.78E-28
rs34006830:34878550:C:A	cg01516881	-13.1824	1.01E-33	1.80E-28
rs17725554:34809907:A:T	cg15383120	-13.1822	1.01E-33	1.80E-28
rs34922677:34460309:C:T	cg11235426	-13.1766	1.07E-33	1.89E-28
rs140754489:34461167:G:A	cg11235426	-13.1766	1.07E-33	1.89E-28
rs12597352:34638507:A:T	cg07332563	-13.175	1.09E-33	1.91E-28
rs7204085:34522955:C:T	cg05064044	-13.1675	1.17E-33	2.05E-28
rs35721740:34708670:G:A	cg11235426	-13.1653	1.19E-33	2.09E-28
rs1433753	cg01516881	-13.1626	1.22E-33	2.13E-28
rs10438601:34625895:C:T	cg05064044	-13.1625	1.22E-33	2.13E-28
rs34728702:34896584:A:G	cg01516881	-13.1483	1.40E-33	2.44E-28
rs56403495:34450108:A:T	cg05064044	-13.1422	1.49E-33	2.57E-28
rs199982235:34463368:T:C	cg05064044	-13.1422	1.49E-33	2.57E-28
rs12445410:34520436:A:C	cg11235426	-13.1393	1.53E-33	2.64E-28
rs11644352:34724788:G:T	cg03395511	-13.0996	2.23E-33	3.84E-28
rs113572057:34190042:G:T	cg11235426	-13.0982	2.26E-33	3.88E-28
rs12445410:34520436:A:C	cg05064044	-13.0946	2.34E-33	4.00E-28
rs11648801	cg01171360	-13.0945	2.34E-33	4.00E-28
rs35721740:34708670:G:A	cg05064044	-13.0806	2.67E-33	4.55E-28
rs35925518:34534674:A:G	cg11235426	-13.0682	3.00E-33	5.11E-28
rs8056602:34693165:G:T	cg21548813	-13.0623	3.17E-33	5.39E-28
rs34922677:34460309:C:T	cg05064044	-13.046	3.70E-33	6.27E-28
rs140754489:34461167:G:A	cg05064044	-13.046	3.70E-33	6.27E-28
rs11646005:35076563:C:T	cg01516881	-13.0425	3.83E-33	6.46E-28
rs55701516:34192380:T:C	cg01171360	-13.0281	4.39E-33	7.39E-28
rs34584967:34475725:T:G	cg01171360	-13.018	4.83E-33	8.12E-28
rs12921999:34197985:G:C	cg01171360	-13.0152	4.96E-33	8.26E-28
rs66873518:34208117:C:T	cg01171360	-13.0152	4.96E-33	8.26E-28
rs17782957:34274796:T:C	cg01171360	-13.0152	4.96E-33	8.26E-28
rs10083785:34279993:A:G	cg01171360	-13.0152	4.96E-33	8.26E-28
rs8056602:34693165:G:T	cg11235426	-12.997	5.89E-33	9.79E-28
rs34584967:34475725:T:G	cg07332563	-12.9965	5.92E-33	9.82E-28
rs12444879:35211534:T:G	cg01516881	-12.9904	6.27E-33	1.04E-27

rs113572057:34190042:G:T	cg05064044	-12.9836	6.69E-33	1.10E-27
rs11648847:35257891:A:T	cg01516881	-12.9808	6.87E-33	1.13E-27
rs17788654:35077080:A:G	cg01516881	-12.9776	7.08E-33	1.16E-27
rs35925518:34534674:A:G	cg05064044	-12.9748	7.27E-33	1.19E-27
rs12923277	cg01516881	-12.9724	7.44E-33	1.22E-27
rs71378081:34345999:TAAAAATAAACA:	cg07332563	-12.9718	7.48E-33	1.22E-27
rs11640596:34347277:T:C	cg07332563	-12.9718	7.48E-33	1.22E-27
rs10438601:34625895:C:T	cg11235426	-12.9698	7.62E-33	1.24E-27
rs35080788:34192617:G:A	cg07332563	-12.9477	9.40E-33	1.52E-27
rs12931636	cg07332563	-12.9443	9.70E-33	1.57E-27
rs8056602:34693165:G:T	cg05064044	-12.9436	9.77E-33	1.58E-27
rs34769185:34654349:G:A	cg03395511	-12.9268	1.15E-32	1.84E-27
rs1973278:34856488:G:A	cg01516881	-12.9197	1.22E-32	1.97E-27
rs76039834:34598453:G:A	cg21548813	-12.9167	1.26E-32	2.02E-27
rs11645488	cg01516881	-12.9141	1.29E-32	2.07E-27
rs8054095:34309883:T:C	cg01171360	-12.8934	1.57E-32	2.51E-27
rs71378081:34345999:TAAAAATAAACA:	cg01171360	-12.893	1.58E-32	2.51E-27
rs11640596:34347277:T:C	cg01171360	-12.893	1.58E-32	2.51E-27
rs8054095:34309883:T:C	cg07332563	-12.8836	1.72E-32	2.73E-27
rs2163975:34884821:C:G	cg01516881	-12.8761	1.85E-32	2.92E-27
rs35080788:34192617:G:A	cg01171360	-12.8434	2.51E-32	3.97E-27
rs12931636	cg01171360	-12.8364	2.69E-32	4.24E-27
rs17723574:34602945:C:G	cg21548813	-12.832	2.80E-32	4.40E-27
rs17725554:34809907:A:T	cg01516881	-12.8243	3.01E-32	4.73E-27
rs12444611:35032947:T:G	cg01516881	-12.7894	4.18E-32	6.55E-27
rs76039834:34598453:G:A	cg05064044	-12.7887	4.21E-32	6.58E-27
rs138201488:35007809:G:GT	cg01516881	-12.778	4.65E-32	7.26E-27
rs8047974	cg07332563	-12.7706	4.99E-32	7.76E-27
rs11647994	cg18110333	-12.7407	6.60E-32	1.03E-26
rs17723574:34602945:C:G	cg05064044	-12.7212	7.93E-32	1.23E-26
rs2163977	cg01516881	-12.6859	1.10E-31	1.71E-26
rs12599106:34498025:T:A	cg03395511	-12.6856	1.11E-31	1.71E-26
rs76039834:34598453:G:A	cg11235426	-12.6782	1.19E-31	1.83E-26
rs8047974	cg01171360	-12.6759	1.21E-31	1.87E-26
rs12597352:34638507:A:T	cg05064044	-12.6687	1.30E-31	1.99E-26
rs12597352:34638507:A:T	cg21548813	-12.6659	1.33E-31	2.04E-26
rs1391904:34548934:C:T	cg21548813	-12.6512	1.53E-31	2.34E-26
rs12933921:34400371:G:C	cg21548813	-12.637	1.75E-31	2.66E-26
rs7195114:46441560:C:T	cg26668828	-12.6358	1.77E-31	2.69E-26
rs12921999:34197985:G:C	cg21548813	-12.6239	1.97E-31	2.98E-26
rs66873518:34208117:C:T	cg21548813	-12.6239	1.97E-31	2.98E-26
rs17782957:34274796:T:C	cg21548813	-12.6239	1.97E-31	2.98E-26
rs10083785:34279993:A:G	cg21548813	-12.6239	1.97E-31	2.98E-26
rs71378663:34532030:T:C	cg03395511	-12.6183	2.08E-31	3.13E-26
rs12149595:35259917:G:A	cg15383120	-12.5936	2.62E-31	3.94E-26
rs2004779:34631340:T:C	cg03395511	-12.5903	2.70E-31	4.05E-26
rs17723574:34602945:C:G	cg11235426	-12.5863	2.80E-31	4.19E-26
rs12926980	cg18110333	-12.578	3.03E-31	4.53E-26
rs55701516:34192380:T:C	cg21548813	-12.5666	3.37E-31	5.02E-26
rs3853177:34498532:A:G	cg21548813	-12.5573	3.67E-31	5.47E-26

rs1391904:34548934:C:T	cg11235426	-12.5275	4.85E-31	7.20E-26
rs56403495:34450108:A:T	cg03395511	-12.506	5.92E-31	8.76E-26
rs199982235:34463368:T:C	cg03395511	-12.506	5.92E-31	8.76E-26
rs7204085:34522955:C:T	cg03395511	-12.5055	5.95E-31	8.79E-26
rs35080788:34192617:G:A	cg21548813	-12.4904	6.85E-31	1.01E-25
rs12921999:34197985:G:C	cg05064044	-12.4699	8.28E-31	1.21E-25
rs66873518:34208117:C:T	cg05064044	-12.4699	8.28E-31	1.21E-25
rs17782957:34274796:T:C	cg05064044	-12.4699	8.28E-31	1.21E-25
rs10083785:34279993:A:G	cg05064044	-12.4699	8.28E-31	1.21E-25
rs1391904:34548934:C:T	cg05064044	-12.4684	8.41E-31	1.22E-25
rs12597352:34638507:A:T	cg11235426	-12.4597	9.11E-31	1.32E-25
rs34922677:34460309:C:T	cg03395511	-12.4503	9.94E-31	1.44E-25
rs140754489:34461167:G:A	cg03395511	-12.4503	9.94E-31	1.44E-25
rs12921999:34197985:G:C	cg11235426	-12.4492	1.00E-30	1.44E-25
rs66873518:34208117:C:T	cg11235426	-12.4492	1.00E-30	1.44E-25
rs17782957:34274796:T:C	cg11235426	-12.4492	1.00E-30	1.44E-25
rs10083785:34279993:A:G	cg11235426	-12.4492	1.00E-30	1.44E-25
rs35721740:34708670:G:A	cg03395511	-12.4283	1.22E-30	1.74E-25
rs12933921:34400371:G:C	cg05064044	-12.4218	1.30E-30	1.85E-25
rs12445410:34520436:A:C	cg03395511	-12.4189	1.33E-30	1.90E-25
rs55701516:34192380:T:C	cg05064044	-12.4179	1.34E-30	1.91E-25
rs55701516:34192380:T:C	cg11235426	-12.4146	1.38E-30	1.97E-25
rs12149595:35259917:G:A	cg01516881	-12.396	1.65E-30	2.33E-25
rs11648801	cg21548813	-12.3929	1.69E-30	2.39E-25
rs35080788:34192617:G:A	cg05064044	-12.3909	1.73E-30	2.43E-25
rs12926980	cg15383120	-12.3899	1.74E-30	2.44E-25
rs3853177:34498532:A:G	cg11235426	-12.3773	1.96E-30	2.74E-25
rs113572057:34190042:G:T	cg03395511	-12.3754	1.99E-30	2.79E-25
rs3853177:34498532:A:G	cg05064044	-12.3739	2.02E-30	2.82E-25
rs76846224:35065215:A:G	cg01516881	-12.3687	2.12E-30	2.95E-25
rs12931636	cg21548813	-12.3684	2.12E-30	2.95E-25
rs35925518:34534674:A:G	cg03395511	-12.3554	2.40E-30	3.33E-25
rs10438601:34625895:C:T	cg03395511	-12.3446	2.65E-30	3.67E-25
rs34584967:34475725:T:G	cg21548813	-12.3445	2.65E-30	3.67E-25
rs11644352:34724788:G:T	cg18110333	-12.3345	2.91E-30	4.01E-25
rs12933921:34400371:G:C	cg11235426	-12.3296	3.04E-30	4.18E-25
rs8056602:34693165:G:T	cg03395511	-12.3182	3.38E-30	4.64E-25
rs8047974	cg21548813	-12.2559	6.01E-30	8.20E-25
rs11647994	cg15383120	-12.2508	6.30E-30	8.58E-25
rs11648801	cg05064044	-12.2467	6.54E-30	8.88E-25
rs35080788:34192617:G:A	cg11235426	-12.2187	8.46E-30	1.14E-24
rs12931636	cg11235426	-12.2124	8.97E-30	1.21E-24
rs11648801	cg11235426	-12.2088	9.26E-30	1.25E-24
rs71378663:34532030:T:C	cg18110333	-12.2086	9.28E-30	1.25E-24
rs12931636	cg05064044	-12.208	9.34E-30	1.25E-24
rs34584967:34475725:T:G	cg05064044	-12.1936	1.07E-29	1.43E-24
rs71378081:34345999:TAAAAATAAACA:[illegible]	cg21548813	-12.152	1.56E-29	2.07E-24
rs11640596:34347277:T:C	cg21548813	-12.152	1.56E-29	2.07E-24
rs34584967:34475725:T:G	cg11235426	-12.1391	1.76E-29	2.33E-24
rs34769185:34654349:G:A	cg18110333	-12.1002	2.51E-29	3.31E-24

rs8047974	cg05064044	-12.0915	2.72E-29	3.58E-24
rs12926980	cg01516881	-12.0881	2.81E-29	3.68E-24
rs8047974	cg11235426	-12.0724	3.24E-29	4.24E-24
rs34922677:34460309:C:T	cg18110333	-12.063	3.53E-29	4.60E-24
rs140754489:34461167:G:A	cg18110333	-12.063	3.53E-29	4.60E-24
rs113572057:34190042:G:T	cg18110333	-12.0568	3.74E-29	4.86E-24
rs11644352:34724788:G:T	cg15383120	-12.0478	4.06E-29	5.27E-24
rs8054095:34309883:T:C	cg21548813	-12.0325	4.66E-29	6.04E-24
rs71378081:34345999:TAAAAATAAACA:T	cg05064044	-12.006	5.95E-29	7.68E-24
rs11640596:34347277:T:C	cg05064044	-12.006	5.95E-29	7.68E-24
rs76039834:34598453:G:A	cg03395511	-11.9831	7.33E-29	9.45E-24
rs71378081:34345999:TAAAAATAAACA:T	cg11235426	-11.967	8.48E-29	1.09E-23
rs11640596:34347277:T:C	cg11235426	-11.967	8.48E-29	1.09E-23
rs8054095:34309883:T:C	cg05064044	-11.9555	9.42E-29	1.21E-23
rs35925518:34534674:A:G	cg18110333	-11.944	1.05E-28	1.34E-23
rs7195114:46441560:C:T	cg07332563	-11.9438	1.05E-28	1.34E-23
rs12599106:34498025:T:A	cg18110333	-11.9192	1.31E-28	1.67E-23
rs17723574:34602945:C:G	cg03395511	-11.9184	1.32E-28	1.68E-23
rs55701516:34192380:T:C	cg03395511	-11.899	1.57E-28	1.99E-23
rs8054095:34309883:T:C	cg11235426	-11.894	1.65E-28	2.08E-23
rs12921999:34197985:G:C	cg03395511	-11.8923	1.67E-28	2.10E-23
rs66873518:34208117:C:T	cg03395511	-11.8923	1.67E-28	2.10E-23
rs17782957:34274796:T:C	cg03395511	-11.8923	1.67E-28	2.10E-23
rs10083785:34279993:A:G	cg03395511	-11.8923	1.67E-28	2.10E-23
rs34769185:34654349:G:A	cg15383120	-11.8802	1.87E-28	2.33E-23
rs11647994	cg01516881	-11.8523	2.41E-28	2.99E-23
rs2004779:34631340:T:C	cg18110333	-11.8104	3.51E-28	4.36E-23
rs11644352:34724788:G:T	cg01516881	-11.7949	4.04E-28	5.00E-23
rs1391904:34548934:C:T	cg03395511	-11.7894	4.25E-28	5.25E-23
rs56403495:34450108:A:T	cg18110333	-11.7655	5.28E-28	6.49E-23
rs199982235:34463368:T:C	cg18110333	-11.7655	5.28E-28	6.49E-23
rs35080788:34192617:G:A	cg03395511	-11.7615	5.47E-28	6.71E-23
rs12597352:34638507:A:T	cg03395511	-11.7582	5.64E-28	6.91E-23
rs7204085:34522955:C:T	cg18110333	-11.7566	5.72E-28	7.00E-23
rs12599106:34498025:T:A	cg15383120	-11.7508	6.02E-28	7.36E-23
rs71378663:34532030:T:C	cg15383120	-11.6785	1.16E-27	1.41E-22
rs12445410:34520436:A:C	cg18110333	-11.6769	1.17E-27	1.43E-22
rs7195114:46441560:C:T	cg01171360	-11.6735	1.21E-27	1.47E-22
rs34769185:34654349:G:A	cg01516881	-11.6579	1.39E-27	1.69E-22
rs12933921:34400371:G:C	cg03395511	-11.6555	1.42E-27	1.72E-22
rs35721740:34708670:G:A	cg18110333	-11.6422	1.60E-27	1.93E-22
rs12921999:34197985:G:C	cg18110333	-11.6418	1.61E-27	1.93E-22
rs66873518:34208117:C:T	cg18110333	-11.6418	1.61E-27	1.93E-22
rs17782957:34274796:T:C	cg18110333	-11.6418	1.61E-27	1.93E-22
rs10083785:34279993:A:G	cg18110333	-11.6418	1.61E-27	1.93E-22
rs55701516:34192380:T:C	cg18110333	-11.6417	1.61E-27	1.93E-22
rs12931636	cg03395511	-11.6158	2.03E-27	2.42E-22
rs10438601:34625895:C:T	cg18110333	-11.6157	2.03E-27	2.42E-22
rs3853177:34498532:A:G	cg03395511	-11.6005	2.33E-27	2.77E-22
rs7204085:34522955:C:T	cg15383120	-11.5874	2.62E-27	3.10E-22

rs56403495:34450108:A:T	cg15383120	-11.5847	2.68E-27	3.17E-22
rs199982235:34463368:T:C	cg15383120	-11.5847	2.68E-27	3.17E-22
rs35080788:34192617:G:A	cg18110333	-11.5786	2.84E-27	3.34E-22
rs8047974	cg03395511	-11.5751	2.92E-27	3.44E-22
rs35721740:34708670:G:A	cg15383120	-11.5406	3.98E-27	4.68E-22
rs2004779:34631340:T:C	cg01516881	-11.5378	4.08E-27	4.79E-22
rs34922677:34460309:C:T	cg15383120	-11.5198	4.80E-27	5.61E-22
rs140754489:34461167:G:A	cg15383120	-11.5198	4.80E-27	5.61E-22
rs11648801	cg03395511	-11.5058	5.44E-27	6.34E-22
rs12445410:34520436:A:C	cg15383120	-11.504	5.52E-27	6.43E-22
rs113572057:34190042:G:T	cg15383120	-11.4933	6.08E-27	7.05E-22
rs2004779:34631340:T:C	cg15383120	-11.4724	7.32E-27	8.46E-22
rs8056602:34693165:G:T	cg18110333	-11.47	7.48E-27	8.62E-22
rs8056602:34693165:G:T	cg15383120	-11.4692	7.53E-27	8.67E-22
rs34584967:34475725:T:G	cg03395511	-11.4409	9.70E-27	1.11E-21
rs71378663:34532030:T:C	cg01516881	-11.4378	9.97E-27	1.14E-21
rs35925518:34534674:A:G	cg15383120	-11.4291	1.08E-26	1.23E-21
rs76039834:34598453:G:A	cg18110333	-11.418	1.19E-26	1.35E-21
rs7195114:46441560:C:T	cg21548813	-11.4084	1.29E-26	1.47E-21
rs12931636	cg18110333	-11.3834	1.62E-26	1.82E-21
rs12599106:34498025:T:A	cg01516881	-11.3725	1.78E-26	2.01E-21
rs35721740:34708670:G:A	cg01516881	-11.3559	2.06E-26	2.32E-21
rs17723574:34602945:C:G	cg18110333	-11.3549	2.08E-26	2.34E-21
rs7195114:46441560:C:T	cg05064044	-11.3491	2.19E-26	2.46E-21
rs10438601:34625895:C:T	cg01516881	-11.3206	2.82E-26	3.14E-21
rs10438601:34625895:C:T	cg15383120	-11.3159	2.94E-26	3.26E-21
rs8047974	cg18110333	-11.2973	3.47E-26	3.84E-21
rs34922677:34460309:C:T	cg01516881	-11.2867	3.81E-26	4.19E-21
rs140754489:34461167:G:A	cg01516881	-11.2867	3.81E-26	4.19E-21
rs71378081:34345999:TAAAAATAAACA:	cg03395511	-11.2578	4.92E-26	5.38E-21
rs11640596:34347277:T:C	cg03395511	-11.2578	4.92E-26	5.38E-21
rs7204085:34522955:C:T	cg01516881	-11.2379	5.86E-26	6.40E-21
rs76039834:34598453:G:A	cg01516881	-11.2331	6.11E-26	6.67E-21
rs35925518:34534674:A:G	cg01516881	-11.2246	6.58E-26	7.17E-21
rs113572057:34190042:G:T	cg01516881	-11.219	6.92E-26	7.49E-21
rs8054095:34309883:T:C	cg03395511	-11.2151	7.16E-26	7.74E-21
rs56403495:34450108:A:T	cg01516881	-11.212	7.36E-26	7.92E-21
rs199982235:34463368:T:C	cg01516881	-11.212	7.36E-26	7.92E-21
rs1391904:34548934:C:T	cg18110333	-11.1928	8.72E-26	9.37E-21
rs7195114:46441560:C:T	cg11235426	-11.1795	9.80E-26	1.05E-20
rs12597352:34638507:A:T	cg18110333	-11.1655	1.11E-25	1.19E-20
rs12933921:34400371:G:C	cg18110333	-11.1605	1.16E-25	1.24E-20
rs12445410:34520436:A:C	cg01516881	-11.1569	1.19E-25	1.27E-20
rs8056602:34693165:G:T	cg01516881	-11.1519	1.25E-25	1.33E-20
rs17723574:34602945:C:G	cg01516881	-11.1242	1.59E-25	1.69E-20
rs3853177:34498532:A:G	cg18110333	-11.0805	2.33E-25	2.47E-20
rs76039834:34598453:G:A	cg15383120	-11.0682	2.60E-25	2.74E-20
rs55701516:34192380:T:C	cg15383120	-11.0621	2.74E-25	2.89E-20
rs12921999:34197985:G:C	cg15383120	-11.0349	3.48E-25	3.64E-20
rs66873518:34208117:C:T	cg15383120	-11.0349	3.48E-25	3.64E-20

rs17782957:34274796:T:C	cg15383120	-11.0349	3.48E-25	3.64E-20
rs10083785:34279993:A:G	cg15383120	-11.0349	3.48E-25	3.64E-20
rs12597352:34638507:A:T	cg01516881	-11.0182	4.02E-25	4.18E-20
rs11648801	cg18110333	-11.013	4.21E-25	4.37E-20
rs1391904:34548934:C:T	cg01516881	-10.9926	5.03E-25	5.20E-20
rs17723574:34602945:C:G	cg15383120	-10.9876	5.25E-25	5.41E-20
rs34584967:34475725:T:G	cg18110333	-10.9506	7.25E-25	7.44E-20
rs7195114:46441560:C:T	cg03395511	-10.9492	7.34E-25	7.52E-20
rs1391904:34548934:C:T	cg15383120	-10.9406	7.90E-25	8.10E-20
rs35080788:34192617:G:A	cg15383120	-10.9248	9.07E-25	9.26E-20
rs12933921:34400371:G:C	cg15383120	-10.9083	1.05E-24	1.07E-19
rs12597352:34638507:A:T	cg15383120	-10.8292	2.08E-24	2.09E-19
rs3853177:34498532:A:G	cg15383120	-10.8152	2.34E-24	2.35E-19
rs8047974	cg15383120	-10.811	2.43E-24	2.43E-19
rs11648801	cg15383120	-10.7977	2.72E-24	2.72E-19
rs71378081:34345999:TAAAAATAAACA:	cg18110333	-10.7721	3.40E-24	3.37E-19
rs11640596:34347277:T:C	cg18110333	-10.7721	3.40E-24	3.37E-19
rs12931636	cg15383120	-10.7657	3.59E-24	3.55E-19
rs12933921:34400371:G:C	cg01516881	-10.7587	3.81E-24	3.77E-19
rs12921999:34197985:G:C	cg01516881	-10.7201	5.31E-24	5.20E-19
rs66873518:34208117:C:T	cg01516881	-10.7201	5.31E-24	5.20E-19
rs17782957:34274796:T:C	cg01516881	-10.7201	5.31E-24	5.20E-19
rs10083785:34279993:A:G	cg01516881	-10.7201	5.31E-24	5.20E-19
rs3853177:34498532:A:G	cg01516881	-10.6794	7.53E-24	7.28E-19
rs8054095:34309883:T:C	cg18110333	-10.6737	7.91E-24	7.61E-19
rs55701516:34192380:T:C	cg01516881	-10.6445	1.02E-23	9.68E-19
rs34584967:34475725:T:G	cg15383120	-10.6194	1.26E-23	1.19E-18
rs35080788:34192617:G:A	cg01516881	-10.5997	1.49E-23	1.40E-18
rs34584967:34475725:T:G	cg01516881	-10.5787	1.78E-23	1.68E-18
rs71378081:34345999:TAAAAATAAACA:	cg15383120	-10.5424	2.43E-23	2.27E-18
rs11640596:34347277:T:C	cg15383120	-10.5424	2.43E-23	2.27E-18
rs12931636	cg01516881	-10.5297	2.70E-23	2.52E-18
rs8054095:34309883:T:C	cg15383120	-10.5287	2.72E-23	2.53E-18
rs7195114:46441560:C:T	cg18110333	-10.5095	3.21E-23	2.98E-18
rs11648801	cg01516881	-10.5044	3.35E-23	3.11E-18
rs8047974	cg01516881	-10.3195	1.60E-22	1.43E-17
rs71378081:34345999:TAAAAATAAACA:	cg01516881	-10.2788	2.25E-22	2.01E-17
rs11640596:34347277:T:C	cg01516881	-10.2788	2.25E-22	2.01E-17
rs8054095:34309883:T:C	cg01516881	-10.1752	5.34E-22	4.71E-17
rs7195114:46441560:C:T	cg15383120	-10.1388	7.23E-22	6.34E-17
rs7195114:46441560:C:T	cg01516881	-9.86068	7.15E-21	6.12E-16

beta	snp_CHR	snp_POS	snp_COUN	snp_ALT	snp_name	snp_numImp
-0.08902	16	34989694	A	G	rs34151874	3
-0.08807	16	35069526	A	G	rs11646602	0
-0.08807	16	35112074	A	G	rs12600198	3
-0.08811	16	34992749	A	G	rs2173885	3
-0.14331	16	35069526	A	G	rs11646602	0
-0.14331	16	35112074	A	G	rs12600198	3
-0.08914	16	34970274	G	C	rs12445057	3
-0.08782	16	34991336	T	C	rs12919333	3
-0.14456	16	34989694	A	G	rs34151874	3
-0.08721	16	35111585	A	G	rs12933929	3
-0.08721	16	35116314	C	G	rs11149549	3
-0.08721	16	35132928	A	T	rs35691226	3
-0.08721	16	35142792	T	G	rs12596272	2
-0.08721	16	35156375	A	G	rs35808007	3
-0.08847	16	34928236	C	T	rs12447240	2
-0.08806	16	34898258	A	C	rs12929704	3
-0.0883	16	34964515	T	A	rs1501462	3
-0.14361	16	34898258	A	C	rs12929704	3
-0.08744	16	34878550	A	C	rs34006830	3
-0.1446	16	34970274	G	C	rs12445057	3
-0.08783	16	34896261	A	G	rs12923277	2
-0.14359	16	34928236	C	T	rs12447240	2
-0.08718	16	34896584	G	A	rs34728702	3
-0.15049	16	34879951	T	C	rs1433753	2
-0.14354	16	34964515	T	A	rs1501462	3
-0.08662	16	35032947	G	T	rs12444611	2
-0.04834	16	34989694	A	G	rs34151874	3
-0.04788	16	35069526	A	G	rs11646602	0
-0.04788	16	35112074	A	G	rs12600198	3
-0.08667	16	35007809	GT	G	rs138201488	3
-0.14123	16	34992749	A	G	rs2173885	3
-0.14293	16	34896261	A	G	rs12923277	2
-0.0913	16	34879951	T	C	rs1433753	2
-0.08921	16	35061015	T	C	rs11645488	1
-0.08549	16	35211534	G	T	rs12444879	3
-0.14019	16	35111585	A	G	rs12933929	3
-0.14019	16	35116314	C	G	rs11149549	3
-0.14019	16	35132928	A	T	rs35691226	3
-0.14019	16	35142792	T	G	rs12596272	2
-0.14019	16	35156375	A	G	rs35808007	3
-0.04848	16	34970274	G	C	rs12445057	3
-0.10991	16	34989694	A	G	rs34151874	3
-0.04761	16	34992749	A	G	rs2173885	3
-0.04769	16	34991336	T	C	rs12919333	3
-0.10866	16	35069526	A	G	rs11646602	0
-0.10866	16	35112074	A	G	rs12600198	3
-0.04734	16	35111585	A	G	rs12933929	3
-0.04734	16	35116314	C	G	rs11149549	3
-0.04734	16	35132928	A	T	rs35691226	3

-0.04734	16	35142792	T	G	rs12596272	2
-0.04734	16	35156375	A	G	rs35808007	3
-0.14811	16	34809907	T	A	rs17725554	3
-0.04802	16	34928236	C	T	rs12447240	2
-0.14033	16	34991336	T	C	rs12919333	3
-0.15002	16	35077080	G	A	rs17788654	3
-0.08605	16	35076563	T	C	rs11646005	3
-0.08915	16	34989694	A	G	rs34151874	3
-0.13906	16	35211534	G	T	rs12444879	3
-0.14065	16	34878550	A	C	rs34006830	3
-0.14497	16	35061015	T	C	rs11645488	1
-0.13987	16	35032947	G	T	rs12444611	2
-0.14043	16	34896584	G	A	rs34728702	3
-0.04786	16	34964515	T	A	rs1501462	3
-0.13989	16	35007809	GT	G	rs138201488	3
-0.10819	16	34992749	A	G	rs2173885	3
-0.10998	16	34970274	G	C	rs12445057	3
-0.04756	16	34898258	A	C	rs12929704	3
-0.08776	16	35069526	A	G	rs11646602	0
-0.08776	16	35112074	A	G	rs12600198	3
-0.10891	16	34928236	C	T	rs12447240	2
-0.10864	16	34898258	A	C	rs12929704	3
-0.10726	16	35111585	A	G	rs12933929	3
-0.10726	16	35116314	C	G	rs11149549	3
-0.10726	16	35132928	A	T	rs35691226	3
-0.10726	16	35142792	T	G	rs12596272	2
-0.10726	16	35156375	A	G	rs35808007	3
-0.11925	16	34989694	A	G	rs34151874	3
-0.08785	16	34992749	A	G	rs2173885	3
-0.04757	16	34896261	A	G	rs12923277	2
-0.10879	16	34964515	T	A	rs1501462	3
-0.13913	16	34856488	A	G	rs1973278	3
-0.0907	16	35077080	G	A	rs17788654	3
-0.11775	16	35069526	A	G	rs11646602	0
-0.11775	16	35112074	A	G	rs12600198	3
-0.08487	16	34884821	G	C	rs2163975	3
-0.04717	16	34878550	A	C	rs34006830	3
-0.10755	16	34991336	T	C	rs12919333	3
-0.0466	16	35211534	G	T	rs12444879	3
-0.08482	16	34856488	A	G	rs1973278	3
-0.04688	16	35032947	G	T	rs12444611	2
-0.13875	16	34884821	G	C	rs2163975	3
-0.08699	16	35111585	A	G	rs12933929	3
-0.08699	16	35116314	C	G	rs11149549	3
-0.08699	16	35132928	A	T	rs35691226	3
-0.08699	16	35142792	T	G	rs12596272	2
-0.08699	16	35156375	A	G	rs35808007	3
-0.08757	16	34991336	T	C	rs12919333	3
-0.0838	16	35257891	T	A	rs11648847	3
-0.13908	16	35076563	T	C	rs11646005	3

-0.08327	16	35065215	G	A	rs76846224	3
-0.04701	16	34896584	G	A	rs34728702	3
-0.10826	16	34896261	A	G	rs12923277	2
-0.04677	16	35007809	GT	G	rs138201488	3
-0.08786	16	34898258	A	C	rs12929704	3
-0.10742	16	34878550	A	C	rs34006830	3
-0.11861	16	34928236	C	T	rs12447240	2
-0.0886	16	34970274	G	C	rs12445057	3
-0.11925	16	34970274	G	C	rs12445057	3
-0.08794	16	34928236	C	T	rs12447240	2
-0.10721	16	34896584	G	A	rs34728702	3
-0.11719	16	34992749	A	G	rs2173885	3
-0.04688	16	35076563	T	C	rs11646005	3
-0.11297	16	34879951	T	C	rs1433753	2
-0.04931	16	34879951	T	C	rs1433753	2
-0.11835	16	34964515	T	A	rs1501462	3
-0.11783	16	34898258	A	C	rs12929704	3
-0.08723	16	34878550	A	C	rs34006830	3
-0.08778	16	34964515	T	A	rs1501462	3
-0.11627	16	35111585	A	G	rs12933929	3
-0.11627	16	35116314	C	G	rs11149549	3
-0.11627	16	35132928	A	T	rs35691226	3
-0.11627	16	35142792	T	G	rs12596272	2
-0.11627	16	35156375	A	G	rs35808007	3
-0.08707	16	34896584	G	A	rs34728702	3
-0.11691	16	34991336	T	C	rs12919333	3
-0.04807	16	35061015	T	C	rs11645488	1
-0.10538	16	35211534	G	T	rs12444879	3
-0.04648	16	34856488	A	G	rs1973278	3
-0.08752	16	34896261	A	G	rs12923277	2
-0.04644	16	34884821	G	C	rs2163975	3
-0.08439	16	34916134	G	A	rs2163977	1
-0.13573	16	35257891	T	A	rs11648847	3
-0.04591	16	35257891	T	A	rs11648847	3
-0.13797	16	34916134	G	A	rs2163977	1
-0.10565	16	35032947	G	T	rs12444611	2
-0.13444	16	35065215	G	A	rs76846224	3
-0.08769	16	34809907	T	A	rs17725554	3
-0.09128	16	34879951	T	C	rs1433753	2
-0.10619	16	35076563	T	C	rs11646005	3
-0.11653	16	34878550	A	C	rs34006830	3
-0.10564	16	35007809	GT	G	rs138201488	3
-0.11722	16	34896261	A	G	rs12923277	2
-0.11632	16	34896584	G	A	rs34728702	3
-0.08536	16	35211534	G	T	rs12444879	3
-0.0485	16	34809907	T	A	rs17725554	3
-0.10908	16	35061015	T	C	rs11645488	1
-0.04927	16	35077080	G	A	rs17788654	3
-0.14437	16	35259917	A	G	rs12149595	3
-0.12253	16	34879951	T	C	rs1433753	2

-0.10547	16	34856488	A	G	rs1973278	3
-0.10531	16	34884821	G	C	rs2163975	3
-0.11237	16	35077080	G	A	rs17788654	3
-0.08561	16	35032947	G	T	rs12444611	2
-0.08567	16	35007809	GT	G	rs138201488	3
-0.08604	16	35076563	T	C	rs11646005	3
-0.04626	16	34916134	G	A	rs2163977	1
-0.08567	16	34856488	A	G	rs1973278	3
-0.08555	16	34884821	G	C	rs2163975	3
-0.04508	16	35065215	G	A	rs76846224	3
-0.11415	16	35211534	G	T	rs12444879	3
-0.0881	16	35061015	T	C	rs11645488	1
-0.11519	16	35076563	T	C	rs11646005	3
-0.10962	16	34809907	T	A	rs17725554	3
-0.08709	16	35259917	A	G	rs12149595	3
-0.115	16	35069526	A	G	rs11646602	0
-0.115	16	35112074	A	G	rs12600198	3
-0.11827	16	35061015	T	C	rs11645488	1
-0.11604	16	34989694	A	G	rs34151874	3
-0.10309	16	35257891	T	A	rs11648847	3
-0.11414	16	35032947	G	T	rs12444611	2
-0.11438	16	34856488	A	G	rs1973278	3
-0.11413	16	35007809	GT	G	rs138201488	3
-0.09056	16	35077080	G	A	rs17788654	3
-0.12198	16	35077080	G	A	rs17788654	3
-0.11494	16	34992749	A	G	rs2173885	3
-0.10475	16	34916134	G	A	rs2163977	1
-0.11416	16	34884821	G	C	rs2163975	3
-0.11652	16	34970274	G	C	rs12445057	3
-0.11474	16	34991336	T	C	rs12919333	3
-0.11386	16	35111585	A	G	rs12933929	3
-0.11386	16	35116314	C	G	rs11149549	3
-0.11386	16	35132928	A	T	rs35691226	3
-0.11386	16	35142792	T	G	rs12596272	2
-0.11386	16	35156375	A	G	rs35808007	3
-0.11253	16	35257891	T	A	rs11648847	3
-0.08498	16	34916134	G	A	rs2163977	1
-0.11542	16	34928236	C	T	rs12447240	2
-0.0885	16	34809907	T	A	rs17725554	3
-0.0833	16	35257891	T	A	rs11648847	3
-0.13077	16	34816462	C	T	rs12926980	0
-0.11915	16	34809907	T	A	rs17725554	3
-0.13066	16	34724788	T	G	rs11644352	3
-0.11526	16	34964515	T	A	rs1501462	3
-0.11474	16	34898258	A	C	rs12929704	3
-0.10109	16	35065215	G	A	rs76846224	3
-0.11367	16	34916134	G	A	rs2163977	1
-0.08144	16	35269324	G	A	rs11647994	1
-0.11391	16	34878550	A	C	rs34006830	3
-0.04745	16	35259917	A	G	rs12149595	3

-0.07918	16	34816462	C	T	rs12926980	0
-0.11359	16	34896584	G	A	rs34728702	3
-0.08195	16	35065215	G	A	rs76846224	3
-0.12998	16	34654349	A	G	rs34769185	3
-0.11964	16	34879951	T	C	rs1433753	2
-0.11416	16	34896261	A	G	rs12923277	2
-0.04372	16	34816462	C	T	rs12926980	0
-0.11186	16	35211534	G	T	rs12444879	3
-0.13229	16	35269324	G	A	rs11647994	1
-0.11211	16	35032947	G	T	rs12444611	2
-0.11276	16	35076563	T	C	rs11646005	3
-0.11206	16	35007809	GT	G	rs138201488	3
-0.10929	16	35065215	G	A	rs76846224	3
-0.1071	16	35259917	A	G	rs12149595	3
-0.12886	16	34498025	A	T	rs12599106	3
-0.13385	16	34532030	C	T	rs71378663	3
-0.11941	16	35077080	G	A	rs17788654	3
-0.11542	16	35061015	T	C	rs11645488	1
-0.04432	16	35269324	G	A	rs11647994	1
-0.1116	16	34856488	A	G	rs1973278	3
-0.11709	16	35259917	A	G	rs12149595	3
-0.11144	16	34884821	G	C	rs2163975	3
-0.11685	16	34989694	A	G	rs34151874	3
-0.07766	16	34724788	T	G	rs11644352	3
-0.11568	16	35069526	A	G	rs11646602	0
-0.11568	16	35112074	A	G	rs12600198	3
-0.12842	16	34631340	C	T	rs2004779	3
-0.12755	16	34522955	T	C	rs7204085	3
-0.12259	16	34879951	T	C	rs1433753	2
-0.12751	16	34450108	T	A	rs56403495	3
-0.12751	16	34463368	C	T	rs199982235	3
-0.04285	16	34724788	T	G	rs11644352	3
-0.13253	16	34460309	T	C	rs34922677	3
-0.13253	16	34461167	A	G	rs140754489	3
-0.12671	16	34520436	C	A	rs12445410	3
-0.13142	16	34190042	T	G	rs113572057	3
-0.10947	16	35257891	T	A	rs11648847	3
-0.12787	16	34625895	T	C	rs10438601	3
-0.08607	16	35259917	A	G	rs12149595	3
-0.13197	16	34534674	G	A	rs35925518	3
-0.11602	16	34809907	T	A	rs17725554	3
-0.11476	16	34992749	A	G	rs2173885	3
-0.11662	16	34970274	G	C	rs12445057	3
-0.11395	16	35111585	A	G	rs12933929	3
-0.11395	16	35116314	C	G	rs11149549	3
-0.11395	16	35132928	A	T	rs35691226	3
-0.11395	16	35142792	T	G	rs12596272	2
-0.11395	16	35156375	A	G	rs35808007	3
-0.09737	16	34816462	C	T	rs12926980	0
-0.11564	16	34928236	C	T	rs12447240	2

-0.11074	16	34916134	G	A	rs2163977	1
-0.12258	16	35077080	G	A	rs17788654	3
-0.11528	16	34898258	A	C	rs12929704	3
-0.09972	16	35269324	G	A	rs11647994	1
-0.10953	16	35269324	G	A	rs11647994	1
-0.08458	16	34989694	A	G	rs34151874	3
-0.08378	16	35069526	A	G	rs11646602	0
-0.08378	16	35112074	A	G	rs12600198	3
-0.07917	16	34816462	C	T	rs12926980	0
-0.0839	16	34992749	A	G	rs2173885	3
-0.11339	16	35211534	G	T	rs12444879	3
-0.11541	16	34964515	T	A	rs1501462	3
-0.12385	16	34708670	A	G	rs35721740	3
-0.12418	16	34598453	A	G	rs76039834	3
-0.07664	16	34654349	A	G	rs34769185	3
-0.0839	16	34991336	T	C	rs12919333	3
-0.11409	16	34991336	T	C	rs12919333	3
-0.1234	16	34602945	G	C	rs17723574	3
-0.07659	16	34498025	A	T	rs12599106	3
-0.10615	16	34816462	C	T	rs12926980	0
-0.10721	16	35065215	G	A	rs76846224	3
-0.08075	16	35269324	G	A	rs11647994	1
-0.08478	16	34970274	G	C	rs12445057	3
-0.08298	16	35111585	A	G	rs12933929	3
-0.08298	16	35116314	C	G	rs11149549	3
-0.08298	16	35132928	A	T	rs35691226	3
-0.08298	16	35142792	T	G	rs12596272	2
-0.08298	16	35156375	A	G	rs35808007	3
-0.04228	16	34654349	A	G	rs34769185	3
-0.11463	16	34896261	A	G	rs12923277	2
-0.09627	16	34724788	T	G	rs11644352	3
-0.04234	16	34498025	A	T	rs12599106	3
-0.11929	16	34809907	T	A	rs17725554	3
-0.1172	16	35061015	T	C	rs11645488	1
-0.11367	16	34878550	A	C	rs34006830	3
-0.11352	16	34896584	G	A	rs34728702	3
-0.0839	16	34928236	C	T	rs12447240	2
-0.12219	16	34638507	T	A	rs12597352	3
-0.0836	16	34898258	A	C	rs12929704	3
-0.12275	16	34693165	T	G	rs8056602	3
-0.11339	16	35076563	T	C	rs11646005	3
-0.07596	16	34450108	T	A	rs56403495	3
-0.07596	16	34463368	C	T	rs199982235	3
-0.07585	16	34522955	T	C	rs7204085	3
-0.08376	16	34964515	T	A	rs1501462	3
-0.12803	16	34197985	C	G	rs12921999	3
-0.12803	16	34208117	T	C	rs66873518	3
-0.12803	16	34274796	C	T	rs17782957	3
-0.12803	16	34279993	G	A	rs10083785	3
-0.04376	16	34532030	C	T	rs71378663	3

-0.0789	16	34532030	C	T	rs71378663	3
-0.07798	16	34724788	T	G	rs11644352	3
-0.11252	16	35032947	G	T	rs12444611	2
-0.12352	16	34400371	C	G	rs12933921	3
-0.12874	16	34192380	C	T	rs55701516	3
-0.07537	16	34520436	C	A	rs12445410	3
-0.11245	16	35007809	GT	G	rs138201488	3
-0.08298	16	34878550	A	C	rs34006830	3
-0.07437	16	34708670	A	G	rs35721740	3
-0.04186	16	34522955	T	C	rs7204085	3
-0.10467	16	34724788	T	G	rs11644352	3
-0.0417	16	34520436	C	A	rs12445410	3
-0.09567	16	34654349	A	G	rs34769185	3
-0.08278	16	34896584	G	A	rs34728702	3
-0.04189	16	34450108	T	A	rs56403495	3
-0.04189	16	34463368	C	T	rs199982235	3
-0.12126	16	34498532	G	A	rs3853177	3
-0.08323	16	34896261	A	G	rs12923277	2
-0.04197	16	34631340	C	T	rs2004779	3
-0.08713	16	34879951	T	C	rs1433753	2
-0.11906	16	34360980	T	C	rs11648801	1
-0.11084	16	35257891	T	A	rs11648847	3
-0.07771	16	34190042	T	G	rs113572057	3
-0.07557	16	34631340	C	T	rs2004779	3
-0.08212	16	35032947	G	T	rs12444611	2
-0.07828	16	34460309	T	C	rs34922677	3
-0.07828	16	34461167	A	G	rs140754489	3
-0.08208	16	35007809	GT	G	rs138201488	3
-0.11342	16	35259917	A	G	rs12149595	3
-0.08139	16	35211534	G	T	rs12444879	3
-0.04333	16	34460309	T	C	rs34922677	3
-0.04333	16	34461167	A	G	rs140754489	3
-0.11172	16	34856488	A	G	rs1973278	3
-0.04091	16	34708670	A	G	rs35721740	3
-0.07394	16	34693165	T	G	rs8056602	3
-0.041	16	34693165	T	G	rs8056602	3
-0.1198	16	34548934	T	C	rs1391904	3
-0.11141	16	34884821	G	C	rs2163975	3
-0.04287	16	34190042	T	G	rs113572057	3
-0.07782	16	34534674	G	A	rs35925518	3
-0.1041	16	34654349	A	G	rs34769185	3
-0.08452	16	35061015	T	C	rs11645488	1
-0.12013	16	34475725	G	T	rs34584967	3
-0.0431	16	34534674	G	A	rs35925518	3
-0.12597	16	34221699	A	C	rs8047974	1
-0.12587	16	34259805	A	G	rs12931636	0
-0.09482	16	34498025	A	T	rs12599106	3
-0.08189	16	35076563	T	C	rs11646005	3
-0.07701	16	34654349	A	G	rs34769185	3
-0.07504	16	34625895	T	C	rs10438601	3

-0.12859	16	34192617	A	G	rs35080788	3
-0.07003	16	34989694	A	G	rs34151874	3
-0.11789	16	34345999	T	TAAAAATA	rs144662970	3
-0.11789	16	34347277	C	T	rs11640596	3
-0.11725	16	35259917	A	G	rs12149595	3
-0.09812	16	34532030	C	T	rs71378663	3
-0.07059	16	34970274	G	C	rs12445057	3
-0.08128	16	34856488	A	G	rs1973278	3
-0.04147	16	34625895	T	C	rs10438601	3
-0.08115	16	34884821	G	C	rs2163975	3
-0.08646	16	35077080	G	A	rs17788654	3
-0.09376	16	34522955	T	C	rs7204085	3
-0.06976	16	34928236	C	T	rs12447240	2
-0.11078	16	34916134	G	A	rs2163977	1
-0.09417	16	34631340	C	T	rs2004779	3
-0.06901	16	34992749	A	G	rs2173885	3
-0.09376	16	34450108	T	A	rs56403495	3
-0.09376	16	34463368	C	T	rs199982235	3
-0.04076	16	34400371	C	G	rs12933921	3
-0.07641	16	34498025	A	T	rs12599106	3
-0.10593	16	35269324	G	A	rs11647994	1
-0.0688	16	35069526	A	G	rs11646602	0
-0.0688	16	35112074	A	G	rs12600198	3
-0.11741	16	34309883	C	T	rs8054095	3
-0.04035	16	34598453	A	G	rs76039834	3
-0.07271	16	34598453	A	G	rs76039834	3
-0.06959	16	34964515	T	A	rs1501462	3
-0.07925	16	34532030	C	T	rs71378663	3
-0.10259	16	34498025	A	T	rs12599106	3
-0.0929	16	34520436	C	A	rs12445410	3
-0.10762	16	35065215	G	A	rs76846224	3
-0.10286	16	34816462	C	T	rs12926980	0
-0.09709	16	34460309	T	C	rs34922677	3
-0.09709	16	34461167	A	G	rs140754489	3
-0.10293	16	34631340	C	T	rs2004779	3
-0.03976	16	34548934	T	C	rs1391904	3
-0.07217	16	34602945	G	C	rs17723574	3
-0.04002	16	34602945	G	C	rs17723574	3
-0.06822	16	35111585	A	G	rs12933929	3
-0.06822	16	35116314	C	G	rs11149549	3
-0.06822	16	35132928	A	T	rs35691226	3
-0.06822	16	35142792	T	G	rs12596272	2
-0.06822	16	35156375	A	G	rs35808007	3
-0.07299	16	34400371	C	G	rs12933921	3
-0.09604	16	34190042	T	G	rs113572057	3
-0.09144	16	34708670	A	G	rs35721740	3
-0.07152	16	34548934	T	C	rs1391904	3
-0.07599	16	34192380	C	T	rs55701516	3
-0.0788	16	35065215	G	A	rs76846224	3
-0.06853	16	34991336	T	C	rs12919333	3

-0.06891	16	34898258	A	C	rs12929704	3
-0.09655	16	34534674	G	A	rs35925518	3
-0.10609	16	34532030	C	T	rs71378663	3
-0.07917	16	35257891	T	A	rs11648847	3
-0.03974	16	34638507	T	A	rs12597352	3
-0.09341	16	34625895	T	C	rs10438601	3
-0.07597	16	34631340	C	T	rs2004779	3
-0.07558	16	34522955	T	C	rs7204085	3
-0.08056	16	34916134	G	A	rs2163977	1
-0.07564	16	34450108	T	A	rs56403495	3
-0.07564	16	34463368	C	T	rs199982235	3
-0.07163	16	34498532	G	A	rs3853177	3
-0.07517	16	34197985	C	G	rs12921999	3
-0.07517	16	34208117	T	C	rs66873518	3
-0.07517	16	34274796	C	T	rs17782957	3
-0.07517	16	34279993	G	A	rs10083785	3
-0.07045	16	34360980	T	C	rs11648801	1
-0.03967	16	34498532	G	A	rs3853177	3
-0.06847	16	34878550	A	C	rs34006830	3
-0.08381	16	34809907	T	A	rs17725554	3
-0.0785	16	34460309	T	C	rs34922677	3
-0.0785	16	34461167	A	G	rs140754489	3
-0.07142	16	34638507	T	A	rs12597352	3
-0.10147	16	34522955	T	C	rs7204085	3
-0.07416	16	34708670	A	G	rs35721740	3
-0.07214	16	34879951	T	C	rs1433753	2
-0.10227	16	34625895	T	C	rs10438601	3
-0.06832	16	34896584	G	A	rs34728702	3
-0.10146	16	34450108	T	A	rs56403495	3
-0.10146	16	34463368	C	T	rs199982235	3
-0.07493	16	34520436	C	A	rs12445410	3
-0.10146	16	34724788	T	G	rs11644352	3
-0.07756	16	34190042	T	G	rs113572057	3
-0.10069	16	34520436	C	A	rs12445410	3
-0.03885	16	34360980	T	C	rs11648801	1
-0.09943	16	34708670	A	G	rs35721740	3
-0.07797	16	34534674	G	A	rs35925518	3
-0.0906	16	34693165	T	G	rs8056602	3
-0.10499	16	34460309	T	C	rs34922677	3
-0.10499	16	34461167	A	G	rs140754489	3
-0.06803	16	35076563	T	C	rs11646005	3
-0.04159	16	34192380	C	T	rs55701516	3
-0.03929	16	34475725	G	T	rs34584967	3
-0.04126	16	34197985	C	G	rs12921999	3
-0.04126	16	34208117	T	C	rs66873518	3
-0.04126	16	34274796	C	T	rs17782957	3
-0.04126	16	34279993	G	A	rs10083785	3
-0.07364	16	34693165	T	G	rs8056602	3
-0.07076	16	34475725	G	T	rs34584967	3
-0.06698	16	35211534	G	T	rs12444879	3

-0.10381	16	34190042	T	G	rs113572057	3
-0.06688	16	35257891	T	A	rs11648847	3
-0.07215	16	35077080	G	A	rs17788654	3
-0.10448	16	34534674	G	A	rs35925518	3
-0.06813	16	34896261	A	G	rs12923277	2
-0.06976	16	34345999	T	TAAAAATA	rs144662970	3
-0.06976	16	34347277	C	T	rs11640596	3
-0.07511	16	34625895	T	C	rs10438601	3
-0.07584	16	34192617	A	G	rs35080788	3
-0.07398	16	34259805	A	G	rs12931636	0
-0.09891	16	34693165	T	G	rs8056602	3
-0.10098	16	34654349	A	G	rs34769185	3
-0.06735	16	34856488	A	G	rs1973278	3
-0.08999	16	34598453	A	G	rs76039834	3
-0.06954	16	35061015	T	C	rs11645488	1
-0.03866	16	34309883	C	T	rs8054095	3
-0.03851	16	34345999	T	TAAAAATA	rs144662970	3
-0.03851	16	34347277	C	T	rs11640596	3
-0.06967	16	34309883	C	T	rs8054095	3
-0.06717	16	34884821	G	C	rs2163975	3
-0.04181	16	34192617	A	G	rs35080788	3
-0.04078	16	34259805	A	G	rs12931636	0
-0.08924	16	34602945	G	C	rs17723574	3
-0.0704	16	34809907	T	A	rs17725554	3
-0.06668	16	35032947	G	T	rs12444611	2
-0.09817	16	34598453	A	G	rs76039834	3
-0.06669	16	35007809	GT	G	rs138201488	3
-0.0733	16	34221699	A	C	rs8047974	1
-0.10665	16	35269324	G	A	rs11647994	1
-0.09744	16	34602945	G	C	rs17723574	3
-0.06696	16	34916134	G	A	rs2163977	1
-0.0998	16	34498025	A	T	rs12599106	3
-0.07242	16	34598453	A	G	rs76039834	3
-0.04043	16	34221699	A	C	rs8047974	1
-0.09683	16	34638507	T	A	rs12597352	3
-0.0881	16	34638507	T	A	rs12597352	3
-0.08769	16	34548934	T	C	rs1391904	3
-0.0893	16	34400371	C	G	rs12933921	3
-0.12094	16	46441560	C	T	rs7195114	3
-0.09235	16	34197985	C	G	rs12921999	3
-0.09235	16	34208117	T	C	rs66873518	3
-0.09235	16	34274796	C	T	rs17782957	3
-0.09235	16	34279993	G	A	rs10083785	3
-0.10341	16	34532030	C	T	rs71378663	3
-0.08176	16	35259917	A	G	rs12149595	3
-0.09967	16	34631340	C	T	rs2004779	3
-0.07178	16	34602945	G	C	rs17723574	3
-0.10297	16	34816462	C	T	rs12926980	0
-0.09272	16	34192380	C	T	rs55701516	3
-0.08763	16	34498532	G	A	rs3853177	3

-0.07102	16	34548934	T	C	rs1391904	3
-0.09879	16	34450108	T	A	rs56403495	3
-0.09879	16	34463368	C	T	rs199982235	3
-0.09866	16	34522955	T	C	rs7204085	3
-0.09376	16	34192617	A	G	rs35080788	3
-0.10056	16	34197985	C	G	rs12921999	3
-0.10056	16	34208117	T	C	rs66873518	3
-0.10056	16	34274796	C	T	rs17782957	3
-0.10056	16	34279993	G	A	rs10083785	3
-0.09533	16	34548934	T	C	rs1391904	3
-0.07101	16	34638507	T	A	rs12597352	3
-0.10243	16	34460309	T	C	rs34922677	3
-0.10243	16	34461167	A	G	rs140754489	3
-0.07456	16	34197985	C	G	rs12921999	3
-0.07456	16	34208117	T	C	rs66873518	3
-0.07456	16	34274796	C	T	rs17782957	3
-0.07456	16	34279993	G	A	rs10083785	3
-0.09669	16	34708670	A	G	rs35721740	3
-0.09689	16	34400371	C	G	rs12933921	3
-0.09779	16	34520436	C	A	rs12445410	3
-0.10099	16	34192380	C	T	rs55701516	3
-0.07496	16	34192380	C	T	rs55701516	3
-0.06925	16	35259917	A	G	rs12149595	3
-0.0854	16	34360980	T	C	rs11648801	1
-0.10242	16	34192617	A	G	rs35080788	3
-0.07487	16	34816462	C	T	rs12926980	0
-0.07073	16	34498532	G	A	rs3853177	3
-0.10118	16	34190042	T	G	rs113572057	3
-0.09525	16	34498532	G	A	rs3853177	3
-0.06417	16	35065215	G	A	rs76846224	3
-0.09082	16	34259805	A	G	rs12931636	0
-0.10176	16	34534674	G	A	rs35925518	3
-0.09851	16	34625895	T	C	rs10438601	3
-0.08649	16	34475725	G	T	rs34584967	3
-0.10143	16	34724788	T	G	rs11644352	3
-0.07153	16	34400371	C	G	rs12933921	3
-0.09628	16	34693165	T	G	rs8056602	3
-0.09026	16	34221699	A	C	rs8047974	1
-0.07618	16	35269324	G	A	rs11647994	1
-0.09302	16	34360980	T	C	rs11648801	1
-0.07524	16	34192617	A	G	rs35080788	3
-0.07339	16	34259805	A	G	rs12931636	0
-0.06889	16	34360980	T	C	rs11648801	1
-0.10543	16	34532030	C	T	rs71378663	3
-0.09883	16	34259805	A	G	rs12931636	0
-0.09418	16	34475725	G	T	rs34584967	3
-0.08438	16	34345999	T	TAAAAATA	rs144662970	3
-0.08438	16	34347277	C	T	rs11640596	3
-0.06968	16	34475725	G	T	rs34584967	3
-0.10047	16	34654349	A	G	rs34769185	3

-0.09818	16	34221699	A	C	rs8047974	1
-0.06298	16	34816462	C	T	rs12926980	0
-0.0728	16	34221699	A	C	rs8047974	1
-0.10452	16	34460309	T	C	rs34922677	3
-0.10452	16	34461167	A	G	rs140754489	3
-0.10367	16	34190042	T	G	rs113572057	3
-0.07328	16	34724788	T	G	rs11644352	3
-0.08406	16	34309883	C	T	rs8054095	3
-0.09188	16	34345999	T	TAAAAATA	rs144662970	3
-0.09188	16	34347277	C	T	rs11640596	3
-0.09443	16	34598453	A	G	rs76039834	3
-0.06804	16	34345999	T	TAAAAATA	rs144662970	3
-0.06804	16	34347277	C	T	rs11640596	3
-0.09193	16	34309883	C	T	rs8054095	3
-0.10365	16	34534674	G	A	rs35925518	3
-0.0708	16	46441560	C	T	rs7195114	3
-0.09954	16	34498025	A	T	rs12599106	3
-0.09371	16	34602945	G	C	rs17723574	3
-0.09875	16	34192380	C	T	rs55701516	3
-0.06798	16	34309883	C	T	rs8054095	3
-0.09798	16	34197985	C	G	rs12921999	3
-0.09798	16	34208117	T	C	rs66873518	3
-0.09798	16	34274796	C	T	rs17782957	3
-0.09798	16	34279993	G	A	rs10083785	3
-0.07286	16	34654349	A	G	rs34769185	3
-0.06367	16	35269324	G	A	rs11647994	1
-0.09926	16	34631340	C	T	rs2004779	3
-0.06179	16	34724788	T	G	rs11644352	3
-0.09228	16	34548934	T	C	rs1391904	3
-0.09859	16	34450108	T	A	rs56403495	3
-0.09859	16	34463368	C	T	rs199982235	3
-0.09942	16	34192617	A	G	rs35080788	3
-0.09245	16	34638507	T	A	rs12597352	3
-0.09841	16	34522955	T	C	rs7204085	3
-0.0724	16	34498025	A	T	rs12599106	3
-0.07496	16	34532030	C	T	rs71378663	3
-0.09753	16	34520436	C	A	rs12445410	3
-0.03859	16	46441560	C	T	rs7195114	3
-0.06154	16	34654349	A	G	rs34769185	3
-0.09323	16	34400371	C	G	rs12933921	3
-0.09616	16	34708670	A	G	rs35721740	3
-0.10072	16	34197985	C	G	rs12921999	3
-0.10072	16	34208117	T	C	rs66873518	3
-0.10072	16	34274796	C	T	rs17782957	3
-0.10072	16	34279993	G	A	rs10083785	3
-0.10146	16	34192380	C	T	rs55701516	3
-0.09608	16	34259805	A	G	rs12931636	0
-0.09829	16	34625895	T	C	rs10438601	3
-0.09159	16	34498532	G	A	rs3853177	3
-0.07156	16	34522955	T	C	rs7204085	3

-0.07163	16	34450108	T	A	rs56403495	3
-0.07163	16	34463368	C	T	rs199982235	3
-0.10264	16	34192617	A	G	rs35080788	3
-0.09589	16	34221699	A	C	rs8047974	1
-0.07023	16	34708670	A	G	rs35721740	3
-0.06143	16	34631340	C	T	rs2004779	3
-0.0742	16	34460309	T	C	rs34922677	3
-0.0742	16	34461167	A	G	rs140754489	3
-0.08956	16	34360980	T	C	rs11648801	1
-0.0709	16	34520436	C	A	rs12445410	3
-0.07349	16	34190042	T	G	rs113572057	3
-0.07138	16	34631340	C	T	rs2004779	3
-0.09529	16	34693165	T	G	rs8056602	3
-0.07006	16	34693165	T	G	rs8056602	3
-0.09058	16	34475725	G	T	rs34584967	3
-0.06321	16	34532030	C	T	rs71378663	3
-0.07369	16	34534674	G	A	rs35925518	3
-0.09508	16	34598453	A	G	rs76039834	3
-0.08678	16	46441560	C	T	rs7195114	3
-0.09884	16	34259805	A	G	rs12931636	0
-0.0605	16	34498025	A	T	rs12599106	3
-0.05944	16	34708670	A	G	rs35721740	3
-0.09434	16	34602945	G	C	rs17723574	3
-0.09497	16	46441560	C	T	rs7195114	3
-0.06071	16	34625895	T	C	rs10438601	3
-0.07083	16	34625895	T	C	rs10438601	3
-0.09832	16	34221699	A	C	rs8047974	1
-0.06259	16	34460309	T	C	rs34922677	3
-0.06259	16	34461167	A	G	rs140754489	3
-0.0883	16	34345999	T	TAAAAATA	rs144662970	3
-0.0883	16	34347277	C	T	rs11640596	3
-0.05989	16	34522955	T	C	rs7204085	3
-0.05916	16	34598453	A	G	rs76039834	3
-0.06226	16	34534674	G	A	rs35925518	3
-0.06181	16	34190042	T	G	rs113572057	3
-0.08836	16	34309883	C	T	rs8054095	3
-0.05985	16	34450108	T	A	rs56403495	3
-0.05985	16	34463368	C	T	rs199982235	3
-0.09262	16	34548934	T	C	rs1391904	3
-0.06968	16	46441560	C	T	rs7195114	3
-0.09281	16	34638507	T	A	rs12597352	3
-0.09419	16	34400371	C	G	rs12933921	3
-0.05933	16	34520436	C	A	rs12445410	3
-0.05874	16	34693165	T	G	rs8056602	3
-0.0585	16	34602945	G	C	rs17723574	3
-0.09235	16	34498532	G	A	rs3853177	3
-0.06824	16	34598453	A	G	rs76039834	3
-0.07171	16	34192380	C	T	rs55701516	3
-0.07105	16	34197985	C	G	rs12921999	3
-0.07105	16	34208117	T	C	rs66873518	3

-0.07105	16	34274796	C	T	rs17782957	3
-0.07105	16	34279993	G	A	rs10083785	3
-0.05787	16	34638507	T	A	rs12597352	3
-0.09044	16	34360980	T	C	rs11648801	1
-0.05754	16	34548934	T	C	rs1391904	3
-0.06761	16	34602945	G	C	rs17723574	3
-0.09146	16	34475725	G	T	rs34584967	3
-0.09323	16	46441560	C	T	rs7195114	3
-0.0669	16	34548934	T	C	rs1391904	3
-0.07213	16	34192617	A	G	rs35080788	3
-0.06803	16	34400371	C	G	rs12933921	3
-0.06662	16	34638507	T	A	rs12597352	3
-0.06662	16	34498532	G	A	rs3853177	3
-0.06984	16	34221699	A	C	rs8047974	1
-0.06547	16	34360980	T	C	rs11648801	1
-0.08911	16	34345999	T	TAAAAATA	rs144662970	3
-0.08911	16	34347277	C	T	rs11640596	3
-0.06956	16	34259805	A	G	rs12931636	0
-0.05766	16	34400371	C	G	rs12933921	3
-0.0595	16	34197985	C	G	rs12921999	3
-0.0595	16	34208117	T	C	rs66873518	3
-0.0595	16	34274796	C	T	rs17782957	3
-0.0595	16	34279993	G	A	rs10083785	3
-0.05652	16	34498532	G	A	rs3853177	3
-0.08879	16	34309883	C	T	rs8054095	3
-0.05961	16	34192380	C	T	rs55701516	3
-0.06563	16	34475725	G	T	rs34584967	3
-0.06035	16	34192617	A	G	rs35080788	3
-0.05607	16	34475725	G	T	rs34584967	3
-0.06441	16	34345999	T	TAAAAATA	rs144662970	3
-0.06441	16	34347277	C	T	rs11640596	3
-0.05857	16	34259805	A	G	rs12931636	0
-0.06458	16	34309883	C	T	rs8054095	3
-0.09428	16	46441560	C	T	rs7195114	3
-0.05489	16	34360980	T	C	rs11648801	1
-0.05766	16	34221699	A	C	rs8047974	1
-0.05408	16	34345999	T	TAAAAATA	rs144662970	3
-0.05408	16	34347277	C	T	rs11640596	3
-0.05383	16	34309883	C	T	rs8054095	3
-0.06735	16	46441560	C	T	rs7195114	3
-0.05642	16	46441560	C	T	rs7195114	3
